# Silicon photomultiplier signal readout and multiplexing techniques for positron emission tomography: a review

**DOI:** 10.1007/s13534-022-00234-y

**Published:** 2022-07-16

**Authors:** Haewook Park, Minseok Yi, Jae Sung Lee

**Affiliations:** 1 Department of Biomedical Sciences, Seoul National University College of Medicine, Seoul, 03080 South Korea; 2 Department of Nuclear Medicine, Seoul National University College of Medicine, 101, Daehak-ro, Jongno-gu, Seoul, 03080 South Korea; 3 Interdisciplinary Program in Bioengineering, Seoul National University College of Engineering, Seoul, 03080 South Korea; 4 Integrated Major in Innovative Medical Science, Seoul National University College of Engineering, Seoul, 03080 South Korea; 5 Brightonix Imaging Inc, Seoul, 04782 South Korea

**Keywords:** Silicon photomultiplier (SiPM), Positron emission tomography (PET), Multiplexing, Time-of-flight (TOF), Radiation detector

## Abstract

In recent years, silicon photomultiplier (SiPM) is replacing the photomultiplier tube (PMT) in positron emission tomography (PET) systems due to its superior properties, such as fast single-photon timing response, small gap between adjacent photosensitive pixels in the array, and insensitivity to magnetic fields. One of the technical challenges when developing SiPM-based PET systems or other position-sensitive radiation detectors is the large number of output channels coming from the SiPM array. Therefore, various signal multiplexing methods have been proposed to reduce the number of output channels and the load on the subsequent data acquisition (DAQ) system. However, the large PN-junction capacitance and quenching resistance of the SiPM yield undesirable resistance–capacitance delay when multiple SiPMs are combined, which subsequently causes the accumulation of dark counts and signal fluctuation of SiPMs. Therefore, without proper SiPM signal handling and processing, the SiPMs may yield worse timing characteristics than the PMTs. This article reviews the evolution of signal readout and multiplexing methods for the SiPM. In this review, we focus primarily on analog electronics for SiPM signal multiplexing, which allows for the reduction of DAQ channels required for the SiPM-based position-sensitive detectors used in PET and other radiation detector systems. Although the applications of most technologies described in the article are not limited to PET systems, the review highlights efforts to improve the physical performance (e.g. spatial, energy, and timing resolutions) of PET detectors and systems.

## Introduction

Positron emission tomography (PET) is a biomedical imaging technique that allows for the quantitative evaluation of the metabolic processes and physiological activities of living bodies by visualizing the distribution of radiolabeled tracers [1–4]. To effectively detect the annihilation photons with relatively high energy of 511 keV, PET scanners are typically ring-shaped and consist of scintillation crystal-based radiation detector modules.

Conventionally, photomultiplier tubes (PMTs) have been widely used for scintillation detectors for radiation measurement and imaging owing to their robustness, high quantum efficiency, and high intrinsic gain [5–14]. However, clinical PET detectors based on PMT arrays have limited intrinsic spatial resolution owing to the large volume of PMTs [15]. In addition, the scintillation light loss due to the gap between the photosensitive areas of PMT array, the low quantum efficiency of the photocathode, and the large transit time jitter of traveling electrons further limit the timing performance of PMT-based PET detectors [16–20].

With the advancement of semiconductor technology, solid-state photosensors have been actively investigated to achieve high photon detection efficiency and signal amplification gain with good noise characteristics suitable for various applications [21–26]. Insensitivity to the magnetic field is one of the advantages of solid-state photosensors; this characteristic has attracted considerable attention from the PET community for developing simultaneous PET/MR imaging systems [27–35]. The avalanche photodiode (APD) is an early-generation solid-state photosensor that detects incoming photons in the depletion region between the P and N-doped semiconductors and has the high intrinsic amplification gain of electrical signals [36]. Also, the APD has attracted interest in various photon detection applications thanks to its small size and low bias voltage operation. However, conventional APDs have an intrinsic disadvantage of low avalanche multiplication gain [37]. Accordingly, the silicon photomultiplier (SiPM), also known as Geiger-mode APD (G-APD), has been developed by interconnecting a large number of small-sized APDs (i.e. single-photon avalanche diodes or SPADs) in parallel and operating them in Geiger-mode with a self-quenching circuit to stop avalanche ionization [38]. The development of SiPM has made it possible to detect extremely weak light at the photon-counting level with high efficiency.

**Fig. 1:**
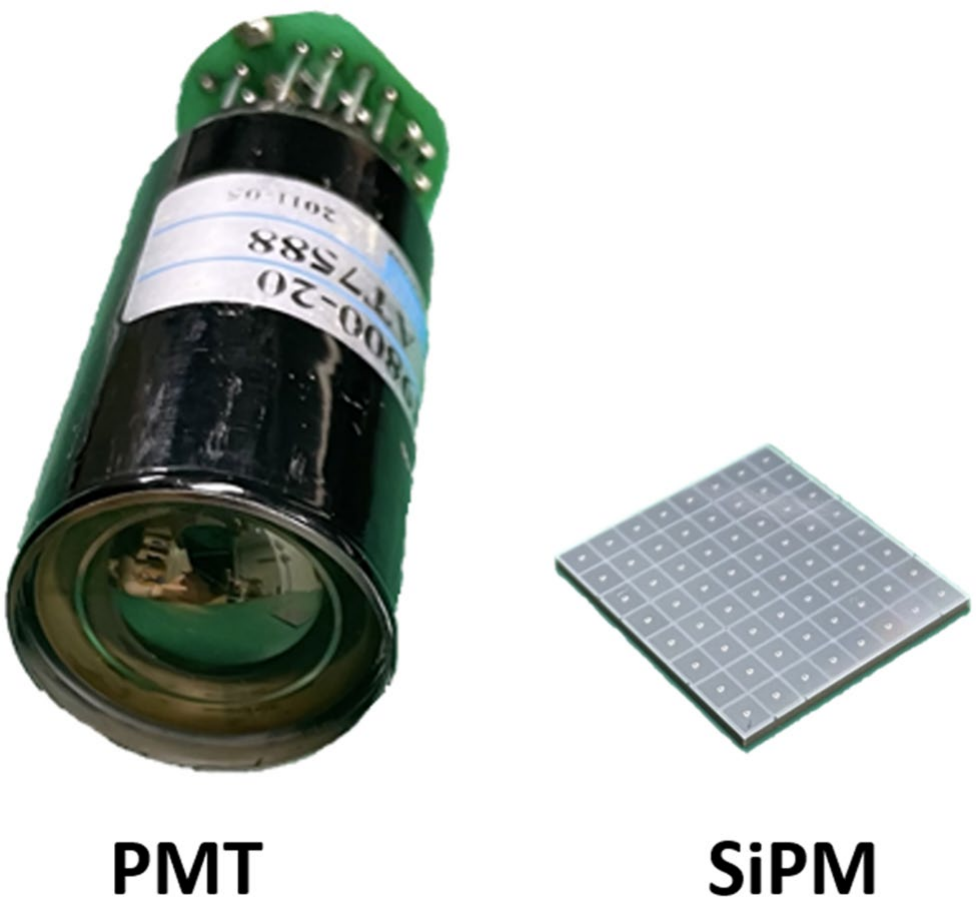
Comparison between a single channel PMT and an 8 × 8 array of SiPM

SiPMs are now replacing PMT in PET systems because of its superior properties, such as fast single-photon timing response, the small gap between adjacent photosensitive pixels in the array, and insensitivity to magnetic fields [33, 34, 37, 39–47] (Fig. 1). Therefore, modern SiPM-based PET scanners offer a better time-of-flight (TOF) capability and a higher spatial resolution than conventional PMT-based PET scanners [15, 48–54].

One of the technical challenges when developing SiPM-based PET systems or other position-sensitive radiation detectors is the large number of output channels coming from the SiPM array. Therefore, various signal multiplexing methods have been proposed to reduce the number of output channels and the load on the subsequent data acquisition (DAQ) system. However, the large PN-junction capacitance and quenching resistance of SiPMs cause undesirable resistance–capacitance (RC) delay when multiple SiPM’s are combined [55]. The combination causes accumulated dark counts that result in signal fluctuation. Therefore, without proper SiPM signal handling and processing, SiPMs may yield worse timing characteristics than PMTs which exhibit low detector capacitance and require no additional quenching resistors.

This article reviews the evolution of signal readout and multiplexing methods for SiPM-based detectors. We focus primarily on analog electronics for SiPM signal multiplexing, which allows for the reduction of DAQ channels that are required for SiPM-based position-sensitive detectors used in PET and other radiation detector systems. The first section reviews the basic principles of PET detector and crystal-photosensor coupling schemes. Next, various SiPM signal multiplexing methods and other technologies to improve the SiPM-based detector performance are introduced. It is worth noting that the signal readout and multiplexing techniques introduced in this review article can also be applied in other systems that utilize SiPM for detecting visible and invisible photons. Although the applications of most technologies described in the article are not limited to PET systems, the review highlights efforts to improve the physical performance (e.g. spatial, energy, and timing resolution) of PET detectors and systems.

## PET detector

### Basic principle

**Fig. 2:**
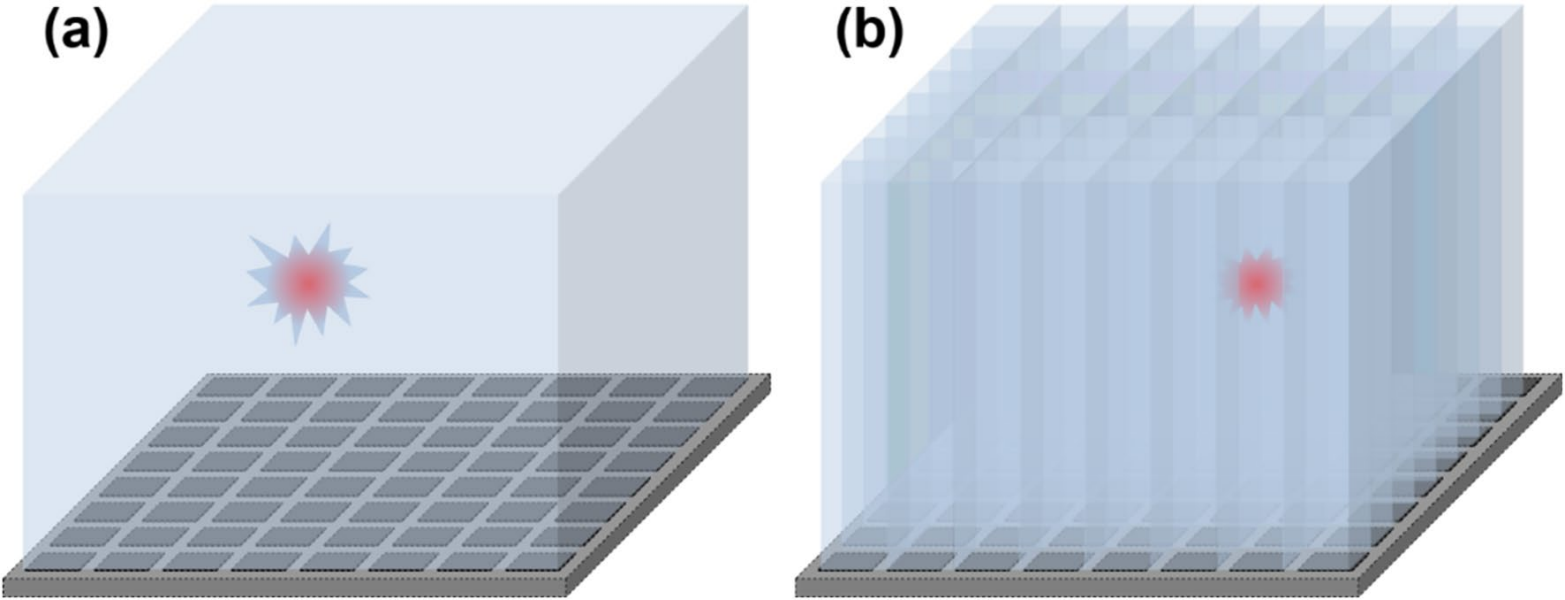
PET detectors with **a**, monolithic crystal and **b** pixelated crystal

A basic element of PET scanners is a detector module assembled with monolithic or pixelated scintillation crystal(s) and photosensor array (Fig. 2). In PET, the origin of radioactive sources is localized by performing image reconstruction that basically superimposes multiple line-of-responses (LORs) or segment-of-responses (SORs) incorporating TOF information. A true LOR (or SOR) is recorded when two 511 keV annihilation photons simultaneously interact with a pair of opposing PET detectors [56]. Based on the output signals from a photosensor array, we can estimate the position of interacted crystals, the arrival time difference, and the deposited energy of annihilation photons. Typically, the PET detector performance is characterized by floodmap quality, energy resolution, and coincidence time resolution (CTR: measurement uncertainty of the arrival time difference between two annihilation photons in terms of the full-width at half-maximum of time difference histogram) which reflect the position, energy, and precision of timing measurement, respectively. The performance of PET detectors is highly dependent on the crystal and photosensor configurations, as well as the front-end circuitry used for reading out the photosensor signals.

### Crystal and SiPM coupling scheme

Fast and bright inorganic scintillation crystals with high density and effective atomic number are used to achieve good detector performance while effectively detecting annihilation photons with a relatively high energy of 511 keV. A sensitivity of PET system can be improved by extending the crystal thickness. However, the longer the crystal, the lower is the CTR performance. The collection efficiency and arrival time jitter of traveling scintillation photons are changed by the crystal surface treatment conditions (i.e. polished or unpolished), significantly affecting PET detector performance.

There are two different schemes for crystal and SiPM coupling: (1) one-to-one coupling and (2) light-sharing configuration. In the one-to-one crystal and SiPM coupling scheme, the dimensions of the crystal and SiPM elements are usually identical, and each crystal element is optically isolated by specular (e.g. enhanced specular reflector or ESR films) or diffusive reflectors (e.g. Teflon tape and BaSO_4_ powder). In the light-sharing configuration, scintillation photons are dispersed in a monolithic crystal block or pixelated crystal array and then measured by multiple SiPMs. Using the light-sharing configuration, the number of DAQ channels in the PET system can be effectively reduced, and the fine intrinsic spatial resolution of the PET detector can reach a size smaller than that of the SiPM pixel. Light guides are typically used in the light-sharing configuration for pixelated crystal arrays to improve crystal positioning accuracy [57–61].

### Photosensor configuration

#### Single-ended readout

**Fig. 3:**
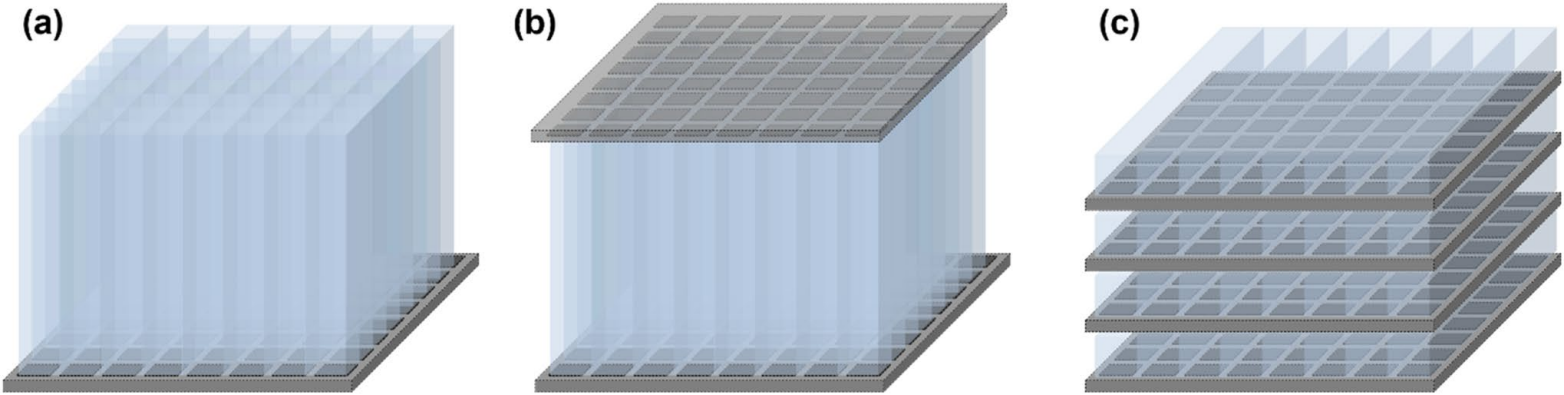
Photosensor configurations for scintillation light readout: **a** single-ended readout, **b** dual-ended readout, and **c** lateral side-readout

A standard approach for measuring scintillation photons emitted from a PET crystal block is to attach SiPM arrays to only the back or front surface of the crystal block, typically referred to as a single-ended readout configuration (Fig. 3a). In the single-ended readout configuration, all crystal block surfaces are covered with reflectors, so that scintillation photons are collected through only a single side of the crystal surface optically coupled with the SiPMs.

#### Dual-ended readout

Another approach is to collect scintillation photons from a PET crystal block using SiPM arrays placed on both (front and back) ends of the crystal block. This method is called a dual-ended readout configuration (Fig. 3b). At the expense of the doubled SiPM cost, the dual-ended readout method makes it possible to measure the depth-of-interaction (DOI) of annihilation photons. The DOI is determined by comparing the amount of scintillation photons collected by SiPMs placed on the opposite sides [62–66]. The DOI measurement enhances the PET image quality by mitigating parallax errors occurring at the periphery of the PET scanner’s transverse field-of-view [67–71]. The timing resolution of PET detectors can be also improved by reducing DOI-dependent time measurement errors [72–76].

#### Lateral side-readout

In the lateral side-readout configuration, a lateral side of each crystal element is covered by SiPMs (Fig. 3c). The lateral side-readout results in the improvement of light collection efficiency and reduction in the transit time variation of scintillation photons; this improves the overall timing performance of PET detectors [77–80]. This method also allows for the 3D measurement of each position of high energy photon interaction (Compton scattering or photo-electric absorption) within a pixelated crystal array, enabling the inter-crystal scatter (ICS) event identification and recovery [81, 82]. The ICS event recovery increases PET system sensitivity and improves image quality [83, 84]. However, the lateral side-readout configuration requires a substantially increased number of photosensors and thus suffers from a high readout complexity and manufacturing costs.

#### Sparse SiPM arrangement

**Fig. 4:**
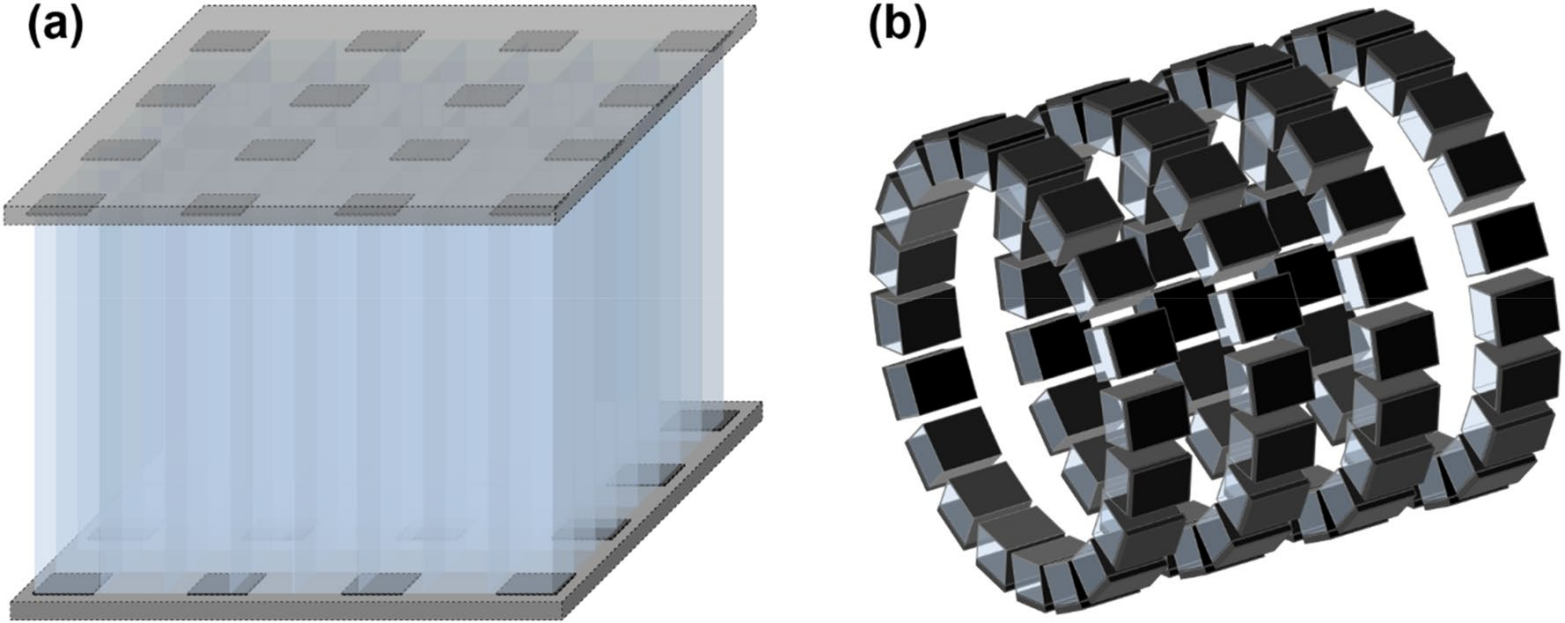
Sparse arrangement of **a** SiPMs and **b** PET detectors

Utilizing sparsely arranged SiPM arrays coupled to scintillation crystal arrays allows the detector manufacturing cost to be reduced, at the expense of light collection efficiency degradation [85] (Fig. 4a). Optimizing the sparse SiPM layout to resolve crystal elements smaller than the SiPM pitch is a major research interest. Detector performance degradation due to the sparse SiPM arrangement should also be minimized.

Recently, there has been a growing interest in sparsely arranged detector configurations for total-body PET applications. The total-body PET scanner provides dynamic PET data for whole-body kinetic analysis and parametric imaging. However, the high material cost for a large number of many scintillation detectors still hinders the widespread use of this new technology. Therefore, various cost-effective long axial field-of-view PET scanner designs based on the sparse arrangement of PET detectors in axial [86–88] or transverse [85, 89] directions have been proposed, which demonstrated the ways to minimize the image quality degradation due to the smaller use of PET detectors (Fig. 4b).

## SiPM signal readout and multiplexing

As mentioned above, SiPM is contributing to improved imaging performance and replacing PMT in modern PET system [15, 33, 48–51, 53, 90–94]. This section introduces SiPM signal readout and multiplexing methods that may be useful for developing SiPM-based PET detectors and systems.

### Individual readout scheme

A simple way to achieve superior SiPM-based PET detector performance is to manage each SiPM signal from the PET detector with an individual readout scheme. In terms of circuitry, the individual readout scheme is simply implemented by connecting the SiPM signals (or pre-amplified SiPM signals) from the PET detector to the subsequent DAQ system. In principle, the individual readout of SiPM signals may lead to the best achievable PET system performance. This is because individual readout allows us to avoid the accumulation of detector capacitance and the baseline fluctuation caused by interference between SiPM channels (e.g. dark noises).

However, the individual readout scheme requires a large number of high-speed DAQ channels. This results in a lack of scalability, which is required to configure a full-ring PET system. The lack of scalability greatly increases the design complexity and manufacturing costs of PET systems. A practical solution for the individual SiPM signal readout is to use application-specific integrated circuits (ASICs) that can individually handle large numbers of SiPM signals with a small footprint and low power consumption [95]. Recently, various research groups developed and evaluated their own ASICs that feature the number of input channels ranging from 8 to 144 [95–117]. For example, the STiC ASIC developed for a multimodal system combining TOF-PET and ultrasound endoscopy (EndoTOFPET-US) [100, 101, 114] yielded an average CTR of 233 ps at the system level for a total of 128 channels. PETA series [104, 105, 108, 111] utilize a charge integration method for energy estimation that covers up to 144 channels in a compact size, being employed for HYPERImage and SUBLIMA projects. A more detailed description of the various ASICs and their usages can be found in Calo et al. [118].

### Multiplexing readout scheme

Signal multiplexing techniques are widely used to reduce the number of DAQ channels required in PET systems. Reducing the readout channel number while minimizing performance degradation is an important research topic in PET hardware development. Multiplexing methods described below were summarized in Table 1.

**Table 1:** Summary of multiplexing schemes

Multiplexing Schemes		Advantages		Disadvantages
Charge modulation-based multiplexing	DPC	Light-sharing readout of crystal array is possible	Simple circuit design	Undesirable RC delay
			Improved timing performance with hybrid DPC network	
	Row-column sum		Undesirable crosstalk between SiPM channels is better avoided	More preamplifiers and readout channels are used than DPC
	Symmetric charge division (SCD)		Further reduced readout circuit output channel number than the row-column summin	A trade-off between readout complexity and PET count-rate performance (e.g. pulse pile-up and dead time)
	Capacitive multiplexing		A faster and more accurate timing performance of PET systems than resistor-based methods	Need for careful circuit design than resistor-based methods
Time modulation-based multiplexing	Simple multiplexing network design High quality multiplexed output signals insignificantly affected by RC delays		High precision time measurement is required Bulky coaxial cables for time delay is not a practical solution	
			A single sinusoid source for each SiPM limits its scalability Effective and fast frequency analysis algorithm is required	
Frequency modulation-based multiplexing	The readout channel can be reduced to a single channel			
Polarity modulation-based multiplexing	50–75% additional channel reduction is possible by polarity modulation		Tradeoff between multiplexing ratio and count rate performance	
Digital modulation-based multiplexing	Early digitization of SiPM signals allow for a better performance by effectively preventing undesirable noise. accumulation FPGA-only signal digitization method reduces the manufacturing cost of DAQ modules		A limited number of available FPGA input/output (I/O) ports should be considered	

#### Charge modulation-based multiplexing

A useful way to reduce the number of readout channels from the PET detector is to modulate the input charge collected from the SiPM arrays based on charge division (or charge sharing) multiplexing networks. The charge division multiplexing network steers the input charge toward output channels, and the amount of input charge is divided by the impedance between the input channel and each of the multiplexed output channels. This allows the interacted crystal position and photon energy information from the PET detectors to be encoded. The charge division multiplexing method can be used not only for one-to-one coupled crystal arrays but also for light-sharing crystal arrays. Typically, the charge division multiplexing network is implemented based on resistive chains, followed by signal shaping and amplification stages at the front-end electronics module.

**Fig. 5:**
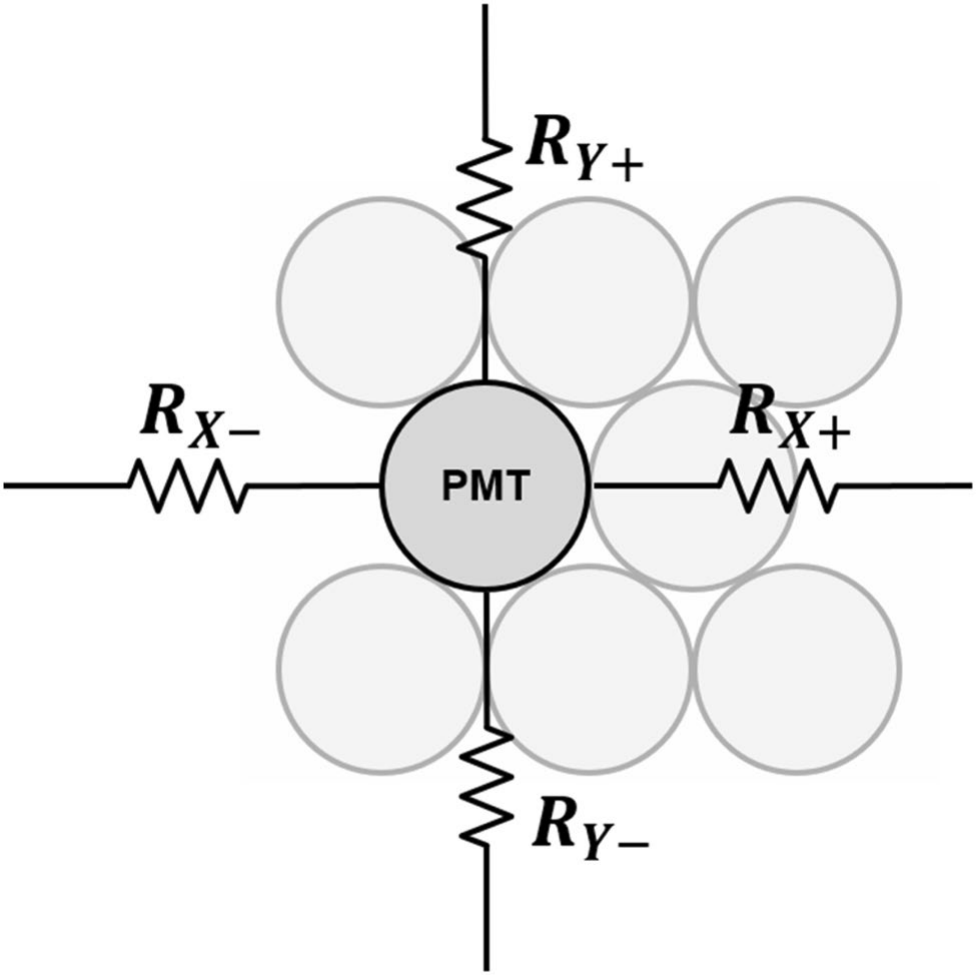
Anger logic circuit

An early version of the charge division multiplexing network was developed by Hal Anger and used in his gamma cameras. In 1958, Anger [119] proposed a position-sensitive readout circuit that reduced the initially large number of PMT array outputs into only four output channels (Fig. 5). He deployed a set of four resistors for each PMT, and the value of resistor sets was chosen to individually encode the interacted position (i.e. Anger logic) within the PMT array. The Anger logic-based multiplexing network is well-established and has shown excellent position decoding accuracy within photosensor arrays [120]. However, it is difficult to implement compact PET detector modules based on the Anger logic-based multiplexing network because such a network requires four passive electronic components for each photosensor elements.

**Fig. 6:**
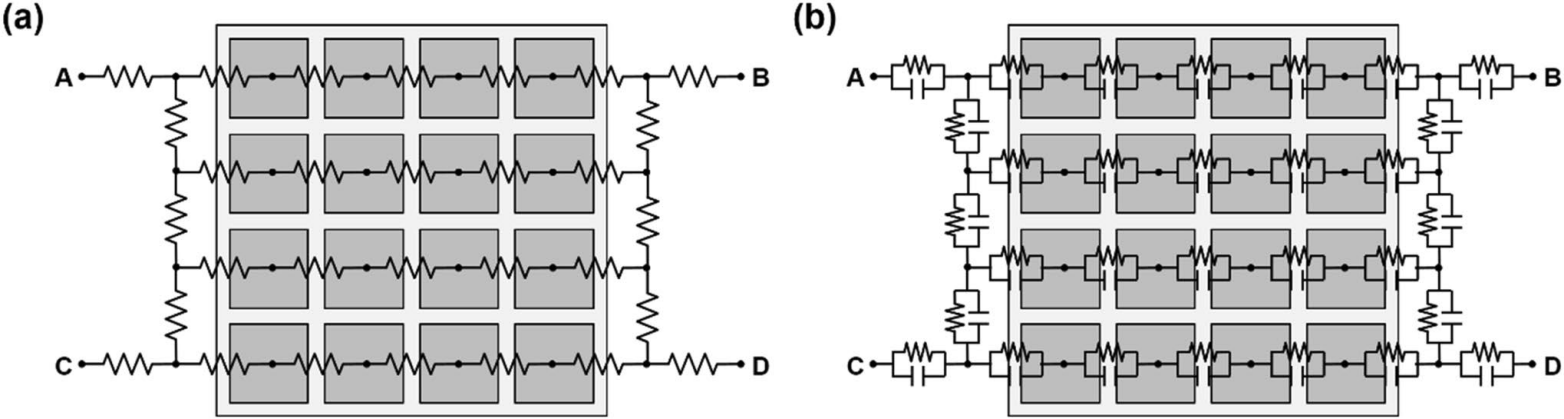
Discretized positioning circuit (DPC): **a** conventional resistive DPC and **b** hybrid DPC using the combination of resistor and capacitor pairs

A more commonly used charge division multiplexing network in PET detectors is the discretized positioning circuit (DPC) [121–130]. The DPC network was originally developed to collect radiation signals from position-sensitive proportional counters in nuclear science applications and was later applied to PET detectors [61, 131]. DPC utilizes a two-dimensional (2D) resistive chain throughout the multiplexing network, where the 2D resistor chain allows the different amounts of input charge to be steered at each DPC node (i.e., the input node of photosensor outputs) toward each of the four output channels (Fig. 6a). In each row, all DPC nodes are connected by a 1D resistor chain. The two-terminal signals of each row are then fed respectively into two 1D resistor chains arranged in a column direction. The split signals by the resister chains in the column direction are collected by four amplifiers at the corner of the multiplexing network. The DPC is also called a four-corner readout circuit because it generates output signals at four corners. The DPC requires fewer passive electronic components than Anger logic to reduce the number of output signals from the photosensor arrays, making it more suitable for developing compact PET detector modules with a simple circuit design. However, unlike PMT-based detectors, DPC combined with a SiPM array suffers from undesirable RC delay in the four-corner output signals due to the relatively large terminal capacitance of SiPM [132]. The RC-filtered SiPM signals by the DPC circuit increase the measurement uncertainty of photon arrival time when the leading-edge discrimination (LED) method is applied [133].

To further improve the performance of the DPC, Park et al*.* [134] investigated and demonstrated a hybrid DPC implemented by cascading the parallel combination of a resistor and capacitor throughout the DPC network (Fig. 6b). Compared with the conventional DPC, the hybrid DPC exhibits an improved timing performance of the PET detector and an excellent pulse shape uniformity that does not depend on the position of the interacted crystals within the PET detectors.

**Fig. 7:**
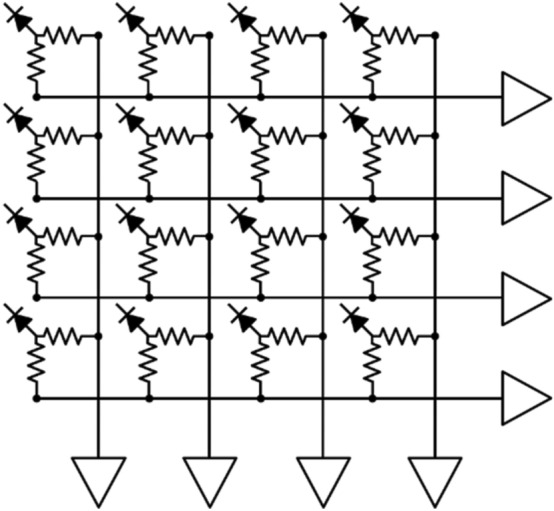
Row-column summing readout circuit

A row-column summing readout circuit is also widely utilized in various PET detectors [135–137]. This features a small form factor when designing the charge division multiplexing network. This makes them suitable for compact high-resolution PET detector implementation. The row-column summing readout circuit is implemented by splitting the SiPM signal (either anode or cathode) into two branches through resistors, capacitors, or diodes (Fig. 7). Subsequently, one branch is used for multiplexing the signals in the row direction of the SiPM array. The other is used for multiplexing the signals in the column direction of the SiPM array. The row-column summing readout circuit features a multiplexing ratio that is lower than that of a DPC network. However, PET detectors based on the row-column summing readout circuits generally outperform DPC-based ones. This is because undesirable crosstalk between SiPM channels is better avoided with row-column summing readout at the expense of using more preamplifiers and readout channels. Recently, an improved row-column summing readout was proposed to minimize the crosstalk between adjacent SiPM channels caused by leakage currents [138]. In this method, the resistors in the conventional row-column summing circuits were replaced by diodes that prevent the charge from flowing back into the adjacent channels by their rectifying function.

**Fig. 8:**
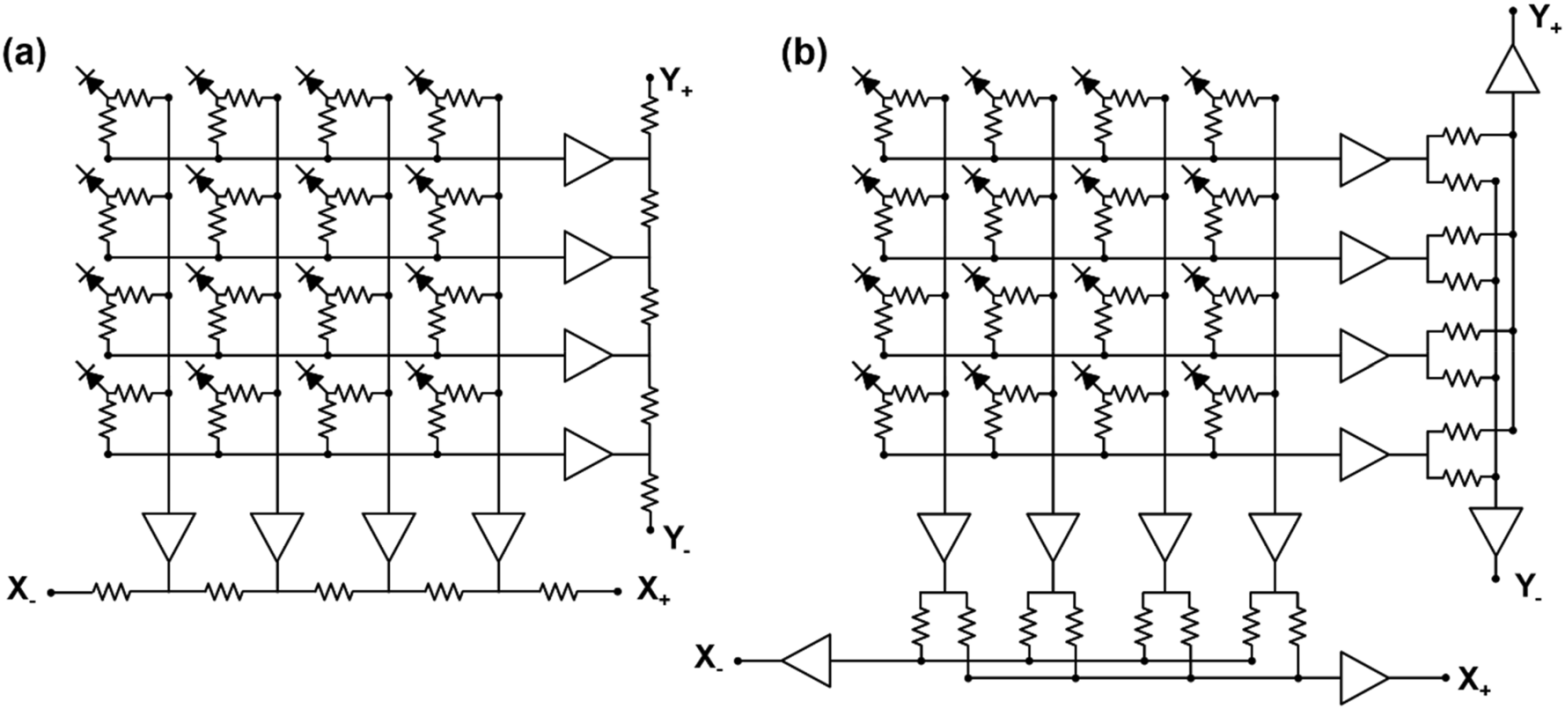
Symmetric charge division (SCD) network using: **a** 1D resistive chains and **b** weighted summing circuits

The symmetric charge division (SCD) network corresponds to a hybrid approach that combines the row-column summing readout circuit and a one-dimensional (1D) resistive chain. Row and column signals from the row-column summing circuit are further multiplexed into four position-encoded signals using 1D resistive chains (Fig. 8a) or weighted summing circuits (Fig. 8b). The additional use of 1D resistive chains or weighted summing circuits further reduces the number of the row-column summing readout circuit output channels; however, there is, a trade-off between readout complexity and PET count-rate performance (e.g. pulse pile-up and dead time) [139–141].

**Fig. 9:**
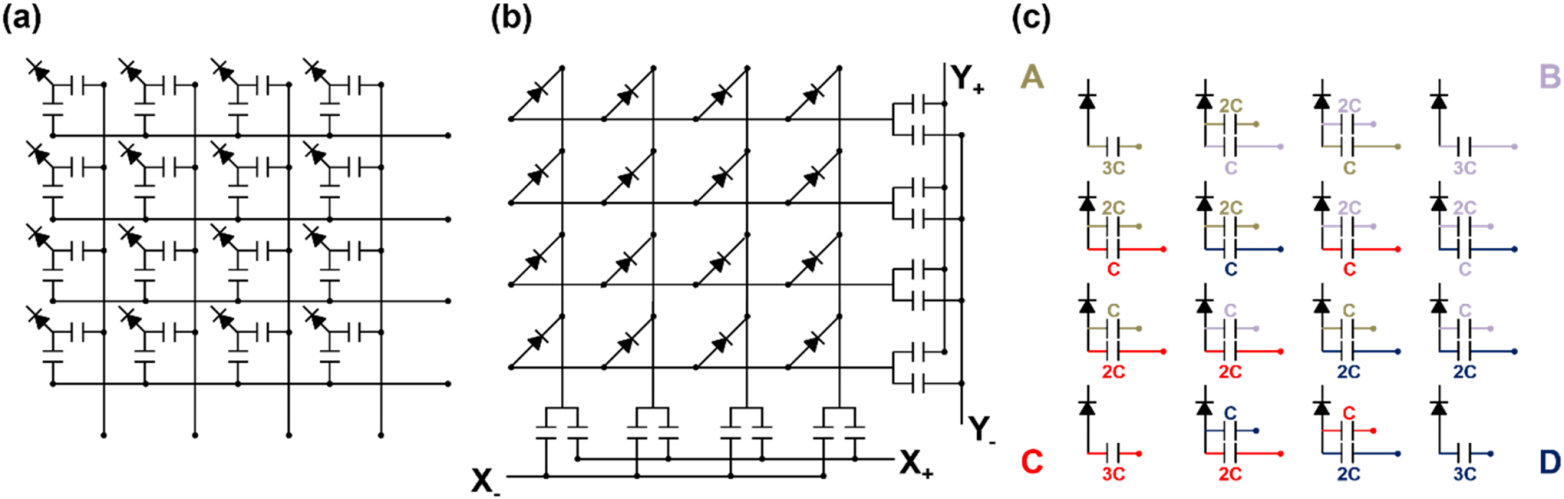
Capacitive multiplexing circuit. **a** Row-column sum, **b** cross-strip capacitive multiplexing, and **c** simplified anger logic

Various studies have been conducted to improve the performance of the charge division multiplexing network by using capacitors instead of resistors. The capacitor-based multiplexing methods have a better high-frequency response. As such, they can achieve a faster and more accurate timing performance of PET systems than resistor-based methods [142]. Sun et al*.* [143] improved the PET detector performance by modifying the conventional row-column summing readout circuit. This was done by simply replacing resistors with capacitors throughout the multiplexing network (Fig. 9a). Olcott et al*.* [144] proposed the concept of cross-strip capacitive multiplexing; each SiPM anode and cathode is individually modulated using linearly-weighted splitting capacitor pairs and summed in the row and column directions, respectively (Fig. 9b). Consequently, the cross-strip capacitive multiplexing network generates four output signals (i.e., two signals for row direction and two signals for column direction) from the SiPM array. Here, the sum of all the independent capacitor pairs for each SiPM element is designed to be constant. Simplified capacitor-based Anger logic methods have been proposed and applied to a 2 × 2 position-sensitive solid-state photomultiplier array and a 4 × 4 SiPM array [145, 146]. In this method, each of detector anodes is split into two branches using a set of weighting capacitors and generates a four-set of position signals by summing the nine-branched signals close to each corner of the photomultiplier array (Fig. 9c).

#### Time modulation-based multiplexing

Signal multiplexing can also be achieved by modulating the time intervals of propagating signals; this is called the time modulation-based multiplexing method. Compared to the charge modulation-based methods, the time modulation-based method typically features a simpler multiplexing network design and yields higher quality multiplexed output signals that are not significantly affected by RC delays as the multiplexing ratio increases. Therefore, time modulation-based multiplexing could be useful for TOF PET detectors because it overcomes the limitation of resistor-based charge division multiplexing methods. In addition, the time modulation-based multiplexing method can be further combined with a time-over-threshold (TOT) technique, which allows for simple energy measurement by recording the time duration above certain thresholds of SiPM signals, thanks to good signal integrity with minimal shape distortion.

**Fig. 10:**
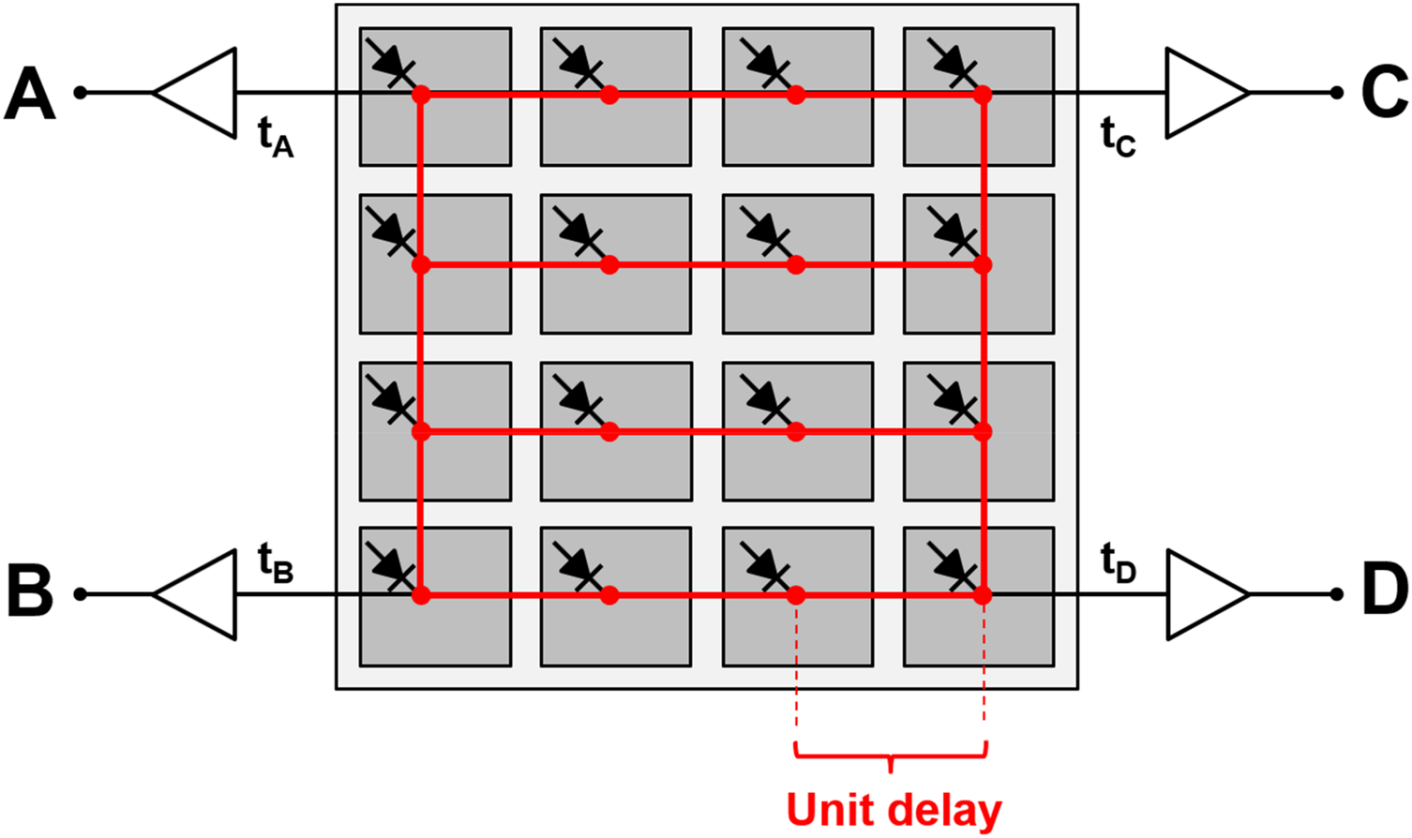
Delay grid multiplexing circuit based on time-modulation

Several useful time modulation-based multiplexing approaches have been proposed. Kim et al*.* [147] proposed a scalable multiplexing solution of SiPM signals using a strip-line readout scheme. The principle of the proposed method is based on measuring the time difference of arrival (TDOA), which is generally used in global positioning system (GPS) applications. The strip-line readout collects two propagating SiPM signals at both ends of signal traces without requiring any passive electronic components for multiplexing SiPM arrays. The position of fired SiPM elements can be uniquely identified using the time difference of two propagated output signals. The non-uniform response of the multiplexed SiPM elements should be considered to achieve a good PET detector performance. In addition, PCB design parameters should be carefully optimized depending on multiplexing ratio. Won et al*.* [148] proposed a 2D TDOA-based multiplexing approach: the so-called delay grid multiplexing method (Fig. 10). The proposed method also does not require any additional passive electronic components for multiplexing SiPM signals and only connects the adjacent signal pins of the SiPM array. Therefore, the delay grid method is implemented in a printed circuit board (PCB) where SiPM elements along the row direction of the SiPM array are connected, and both ends of the row traces are subsequently connected to the two column traces. The fired SiPM element can be identified by measuring the time difference of signal propagation from the SiPM element to each readout channel at the corners. Similar to the strip-line multiplexing method mentioned above, PCB design parameters, including the PCB materials, the dielectric constant, the width of the signal trace, and the PCB height width with respect to the reference plane, should be carefully optimized.

**Fig. 11:**
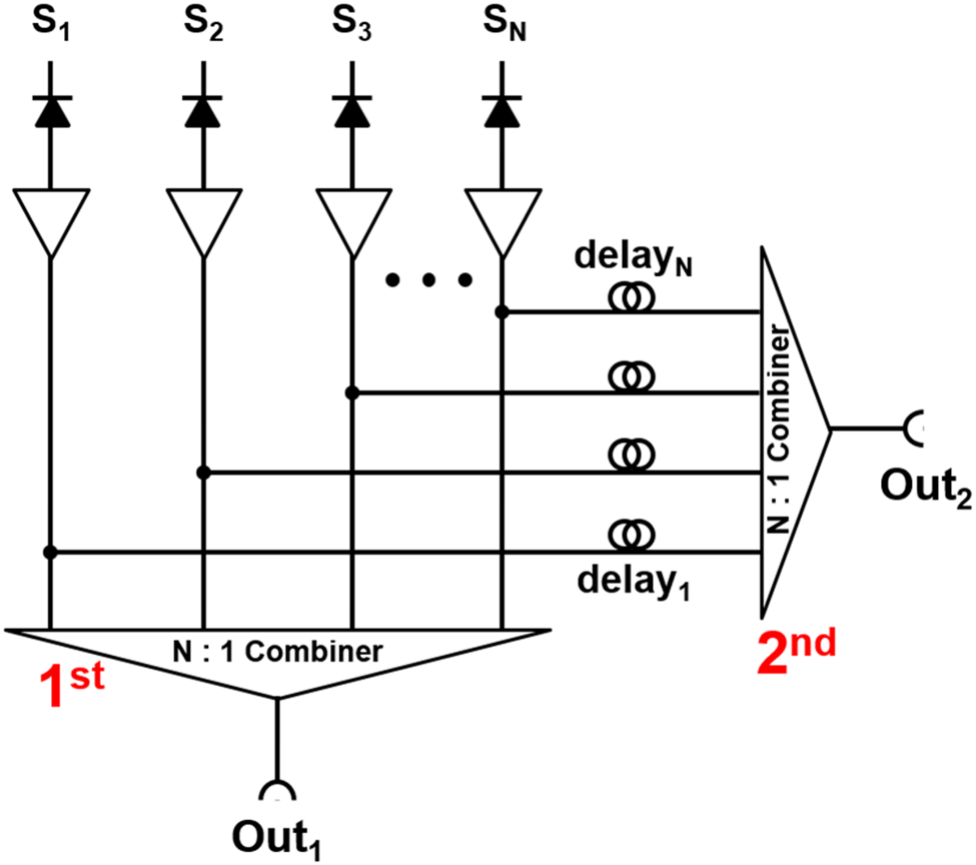
Delay line multiplexing circuit using 2-branch splitting and signal combiners

Vinke et al*.* [149] proposed an electrical delay line multiplexing method (Fig. 11). In this method, the SiPM signal is split into two branches using an RF splitter. One branch is connected to an N-to-1 RF combiner (i.e. first multiplexer), and the other branch is fed into another N-to-1 RF combiner (i.e. second multiplexer). The input signals to the second multiplexer have different time delays. The fired SiPM element in PET detectors is identified by measuring the arrival time difference between two multiplexed output signals. The proposed method can be also used for identifying ICS events by arranging the multiplexing network in a checkerboard pattern, but the further demonstration is required. The trigger level of SiPM signals should be chosen carefully because it affects the performance of SiPM identification and timing performance of PET detectors. The use of coaxial cables is not a practical solution for time delays because of their bulkiness. Therefore, delay chips are used in the subsequent investigations based on the delay line multiplexing methods [81, 84, 150].

#### Frequency modulation-based multiplexing

**Fig. 12:**
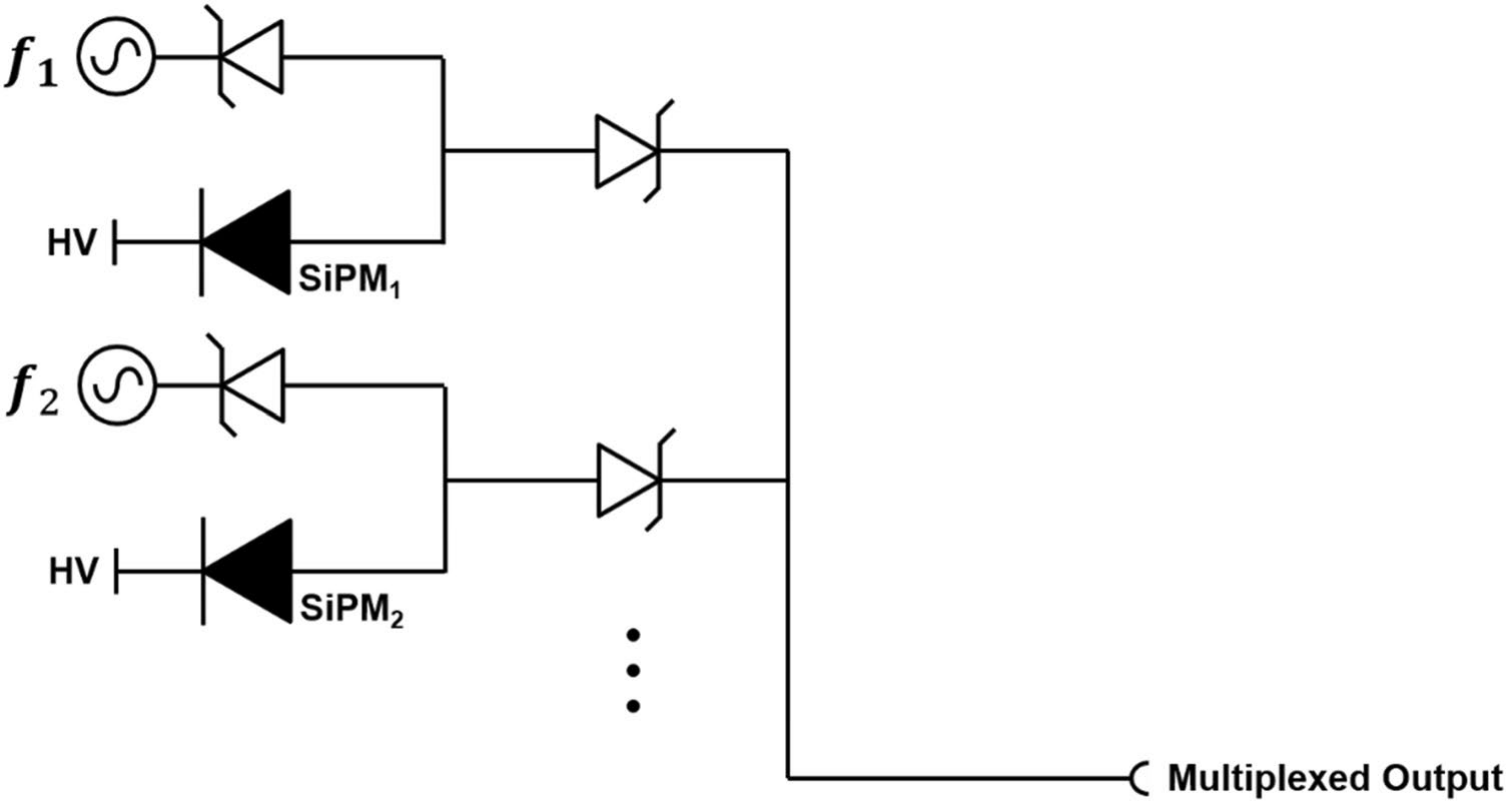
Schottky diode-based frequency modulation-based multiplexing circuit

Frequency modulation techniques are widely used in various applications, including computing, telecommunication, radio broadcasting, and radiation detecting fields [151]. The frequency modulation typically uses a carrier wave with varying frequency to encode information-of-interest. Wonders et al*.* [152] introduced a novel multiplexing method for SiPM-based PET detectors that utilize mixed sinusoidal waves. The proposed multiplexing method requires a single sinusoid source per each SiPM channel and a pair of Schottky diodes to prevent signal interference between the multiplexed SiPM signals (Fig. 12). The sinusoid pulse serves as a tagging signal and is used for pulse shape discrimination. The initial energy and timing performances of the proposed method were demonstrated using a PET-like coincidence measurement setup, showing a potential to be used for SiPM-based PET systems. Although this method allows the readout of multiple SiPMs using a single channel, the requirement for a single sinusoid source for each SiPM limits its scalability. Another major drawback of this multiplexing method is that about half of the signal is lost while splitting the charge between the two diodes. If an adequate modulation to the effective impedance to steer more charge to the forward direction is applied, this method can be one of promising multiplexing methods with high multiplexing ratio.

#### Polarity modulation-based multiplexing

**Fig. 13:**
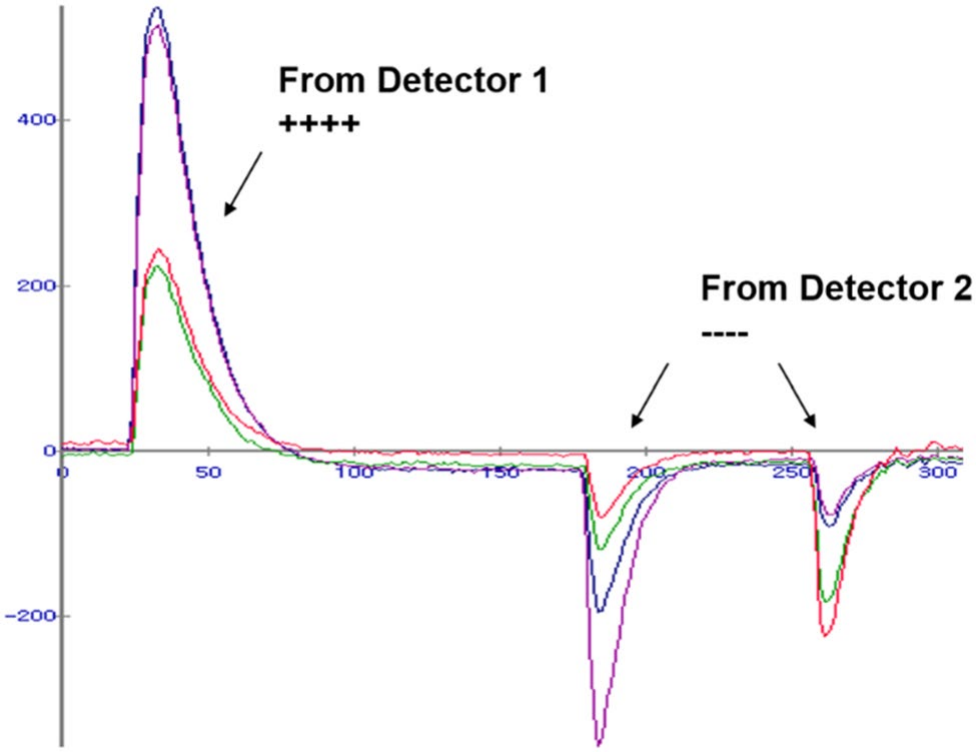
Bipolar multiplexing (Reprint from [153] with permission; © 2014 Institute of Physics and Engineering in Medicine)

**Fig. 14:**
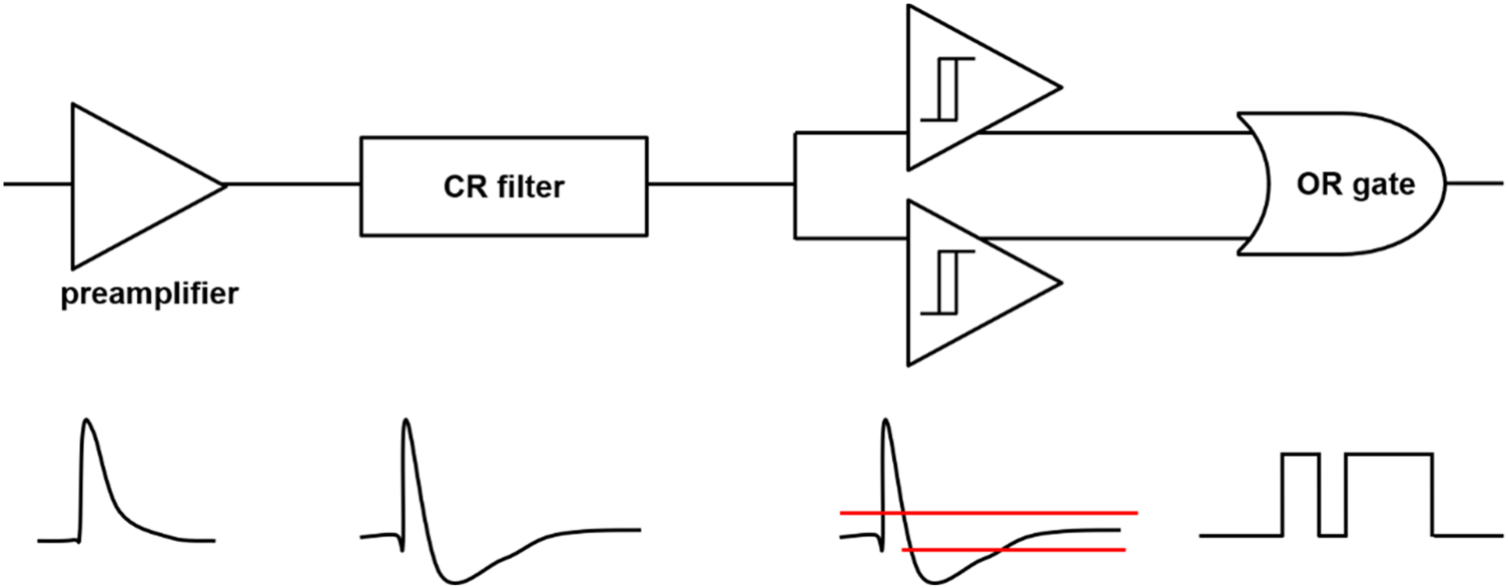
TOT method using bipolar signals

There are multiplexing approaches that modulate PET data based on the polarity of SiPM signals. An analog bipolar multiplexing method was proposed by Yoon et al*.* [153] to further reduce the readout burden on a DAQ system for DPC output signals. In this approach, four DPC output signals were further encoded with different polarity combinations. The proposed method requires only four differential amplifiers and one summing amplifier for the multiple DPC-based PET detectors before the multiplexed signals are fed to the subsequent DAQ system (Fig. 13). The proposed method achieves a 50–75% channel reduction in the DAQ module, depending on a multiplexing ratio [33, 35]. The multiplexing ratio of this approach should be carefully chosen considering the tradeoff between the multiplexing ratio and the PET count rate performance. The polarity modulation can be also applied to the TOT circuit [154]. In this approach, each of four multiplexed output signals generated from the DPC network is subsequently converted into a bipolar signal using a preamplifier, an active capacitor-resistor (CR)-shaping filter, two comparators, and an OR logic gate (Fig. 14). The proposed method improved the energy linearity of the conventional TOT method and yielded a performance similar to the data collection based on free-running analog-to-digital converters (ADCs). Careful parameter optimization of the CR-shaping filter and threshold sweep would be required to achieve optimal PET detector performance at a system level.

#### Digital modulation-based multiplexing

Early digitization of SiPM signals may allow for a better performance of the SiPM-based PET system by effectively preventing the accumulation of undesirable noises (i.e., dark count, after pulse, and optical crosstalk) and interference between the multiplexed SiPM elements [155–157]. In addition, the digital multiplexing based on field-programmable gate array (FPGA)-only signal digitization method [141, 158, 159] reduces the manufacturing cost of DAQ modules by eliminating waveform sampling-based DAQ that uses ADCs. However, a limited number of available FPGA input/output (I/O) ports should be considered when developing the DAQ module that incorporates the FPGA-only signal digitization method.

**Fig. 15:**
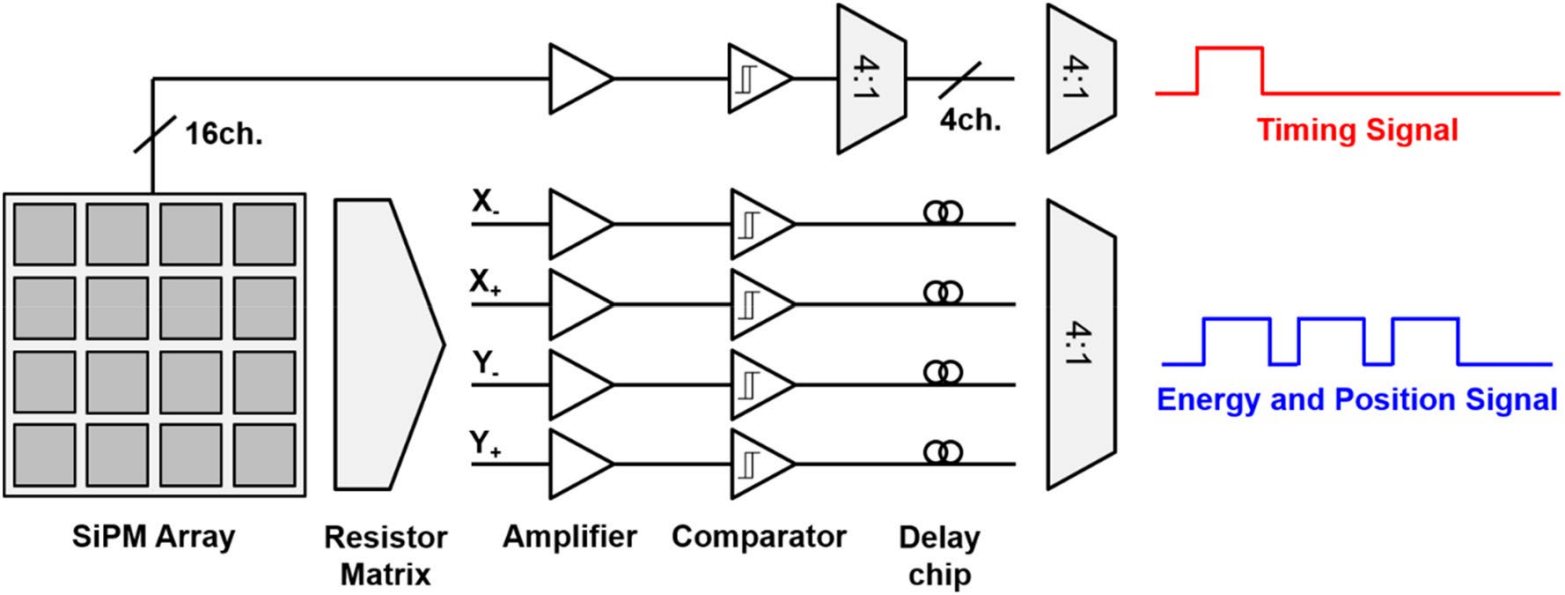
Digital modulation-based multiplexing using active delay chips

The usefulness of digital SiPM multiplexing for PET detectors has been demonstrated in several studies. A scalable multiplexing method based on digital pulse sequence generation was proposed by Cates et al*.* [160] with the goal of reducing DAQ channels while maintaining good timing performance. In this method, 16 SiPM signals are multiplexed into 2 output channels: one provides timing information with fast comparators, and the other generates a digital pulse sequence. The digital pulse sequence is generated using delay chips for both energy and position information encoding (Fig. 15). For energy and position encoding, 16 signals from a 4 × 4 SiPM array were reduced to 4 Anger logic outputs, which were passed to comparators to encode energy information into TOT pulses. TOT pulses were then combined into a single readout line after delaying 3 TOT pulses using active delay chips with an increasing delay in 500 ns increments. The energy was estimated by summing the total width of the digital pulse sequence based on a TOT method. The proposed method achieves the multiplexing ratio of 16:2.

**Fig. 16:**
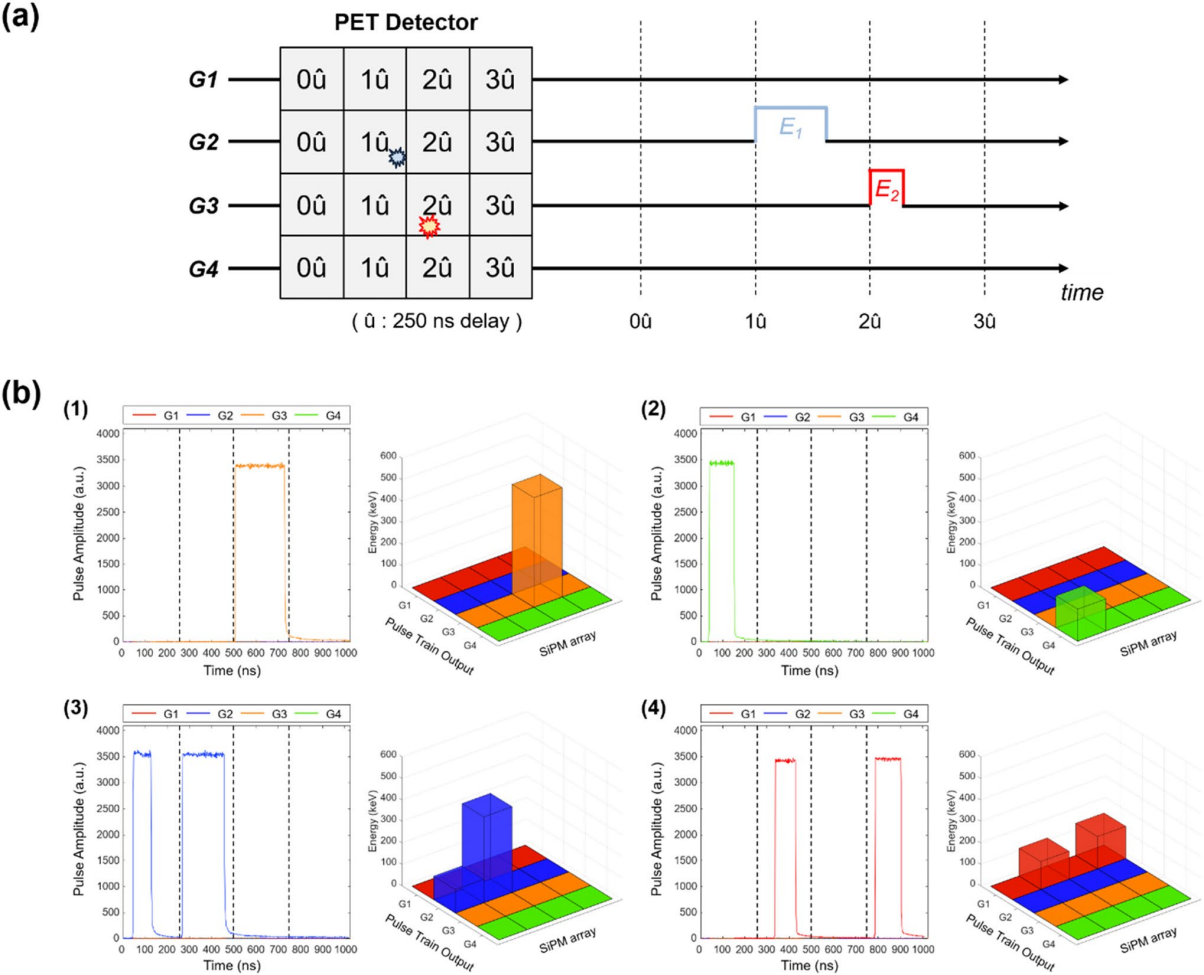
Digital delay encoding method for ICS event identification. **a** Concept illustration. **b** Representative signals from the pulse train outputs: (1) PE absorption event (2) scatter/escape event (3) ICS event, and (4) ICS/escape event (Reprint from [84] with permission; © 2020 Institute of Physics and Engineering in Medicine)

The digital delay encoding technique was also useful in the development of a PET detector with ICS event identification capability [84]. The proposed method serializes 16 one-to-one coupled SiPM anode signals into 4 digital pulse sequences, in which time delays in 250-ns increment were introduced (Fig. 16). Energy information was estimated by the TOT method and position information was decoded by analyzing pre-defined time delays and pulse train output channels. A nearly fourfold reduction of readout channels was achieved for a 4 × 4 PET detector module while maintaining the ICS event identification capability of an individual signal readout scheme with TOF capability.

**Fig. 17:**
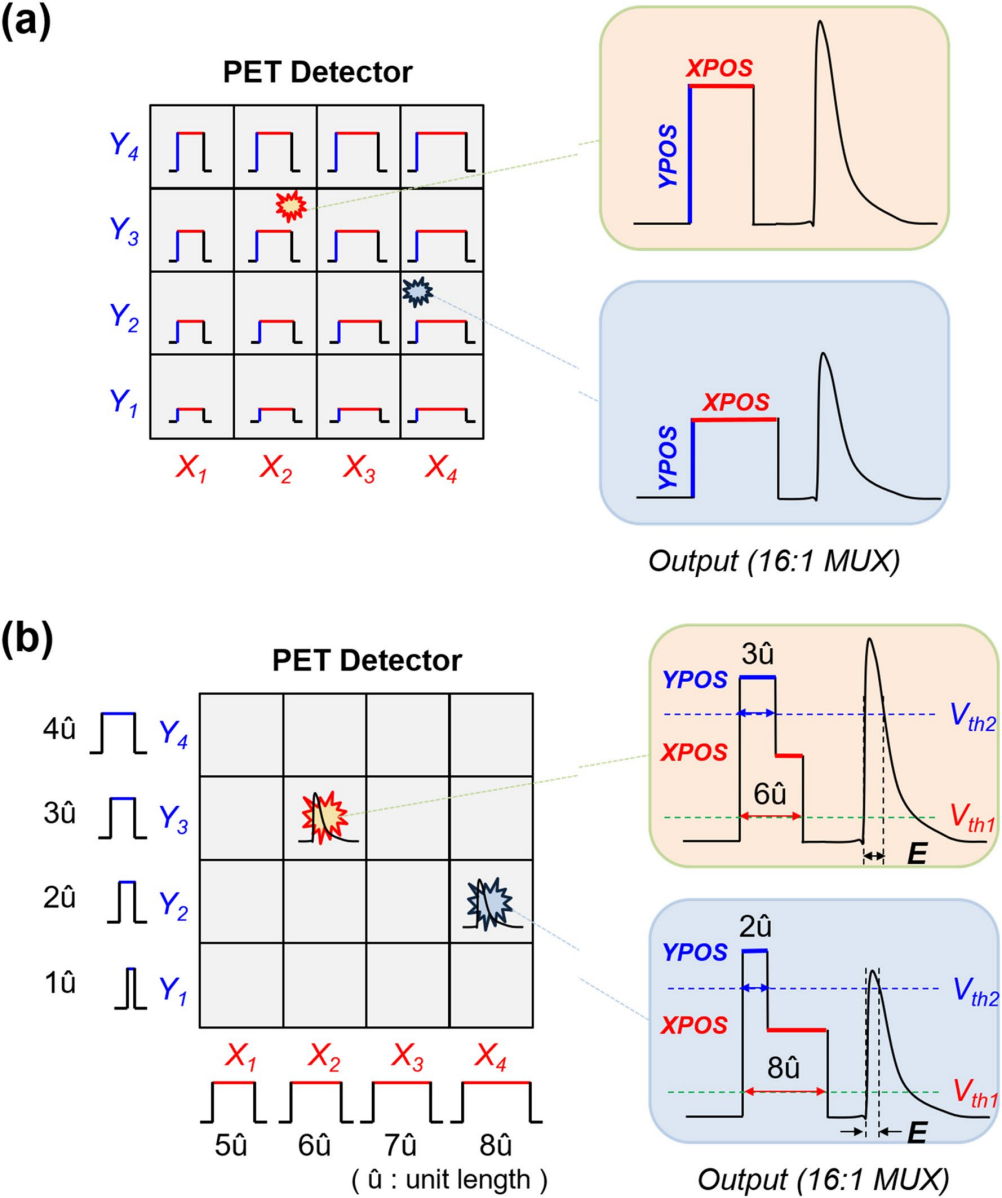
Pulse-tagging multiplexing using **a** rectangular and **b** L-shaped tagging pulses (Reprint from [165] with permission; © Korean Society of Medical and Biological Engineering 2022)

The pulse-tagging method proposed for a single transmission-line readout is a kind of semi-digital multiplexing approach that allows for readout channel reduction without compromising detector performance [161]. In this method, a square tagging pulse is attached ahead of an analog SiPM signal. The position information of the PET detector is encoded into a specific width and height of the tag signal, and timing information is extracted from the rising edge of the tag signal (Fig. 17a). This method requires only a single readout channel to acquire data from a 4 × 4 PET detector module without degrading timing performance as the multiplexing ratio increases. Encoding DOI information with phoswich scintillation crystals [70, 162–164] is also possible with this approach because it preserves the pulse shape information in the multiplexed output signals. Recently, a fully time-based single transmission-line readout method has been also proposed [165]. In this method, an L-shaped tagging pulse was used instead of the square pulse, and a 2D gamma-ray interaction position was encoded in the upper and bottom widths of the L-shaped tag (Fig. 17b). The dual-threshold TOT method applied to the tagged SiPM pulses allows for the simultaneous estimation of position and energy only based on time measurements.

## Other technologies to improve readout performance

### Temperature compensation

**Fig. 18:**
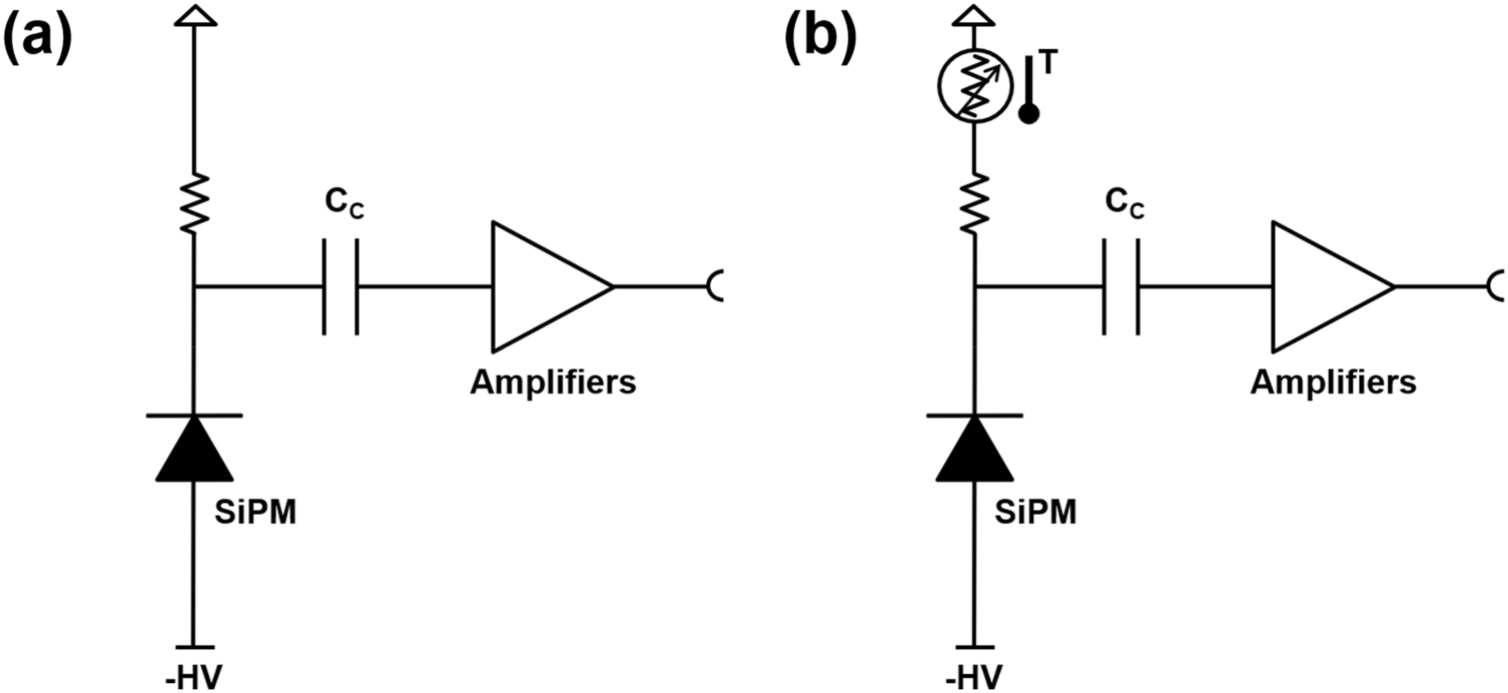
Automatic gain drift-compensation for SiPM: **a** conventional SiPM biasing and **b** SiPM biasing using a temperature sensor with current driving capability

One of the technical challenges of maintaining the performance of SiPM-based PET detectors and systems is the temperature-dependent gain variation of SiPM. Various temperature compensation methods have been proposed because the breakdown voltage and intrinsic gain of the SiPM drift with the changes in the operating temperature [166]. Bias voltage modulation technique which is based on temperature-voltage lookup tables (LUTs) implemented in the FPGA, microcontroller unit, or personal computer is a widely used method [50, 167, 168]. Although this method allows for an accurate gain drift compensation in real time, constructing a LUT for each SIPM or multiplexed SiPM array is a time-consuming and laborious task. Therefore, several automatic gain drift compensation methods that do not require LUTs have also been proposed. Licciulli et al. [169, 170] proposed an automatic compensation method using a dark pulse amplitude of a “blind” SiPM, which should be approximately proportional to the gain of the operating SiPM. In this method, owing to the negative feedback configuration, it is possible to achieve constant SiPM gain without an accurate knowledge of the detector sensitivity to temperature variation; however, this is at the expense of an additional SiPM. Application specific customized temperature sensors based on thermometers and p-n diodes were also developed for the automatic gain drift compensation with LUT [171, 172]**.** These sensors provide output voltage linearly proportional to the temperature, allowing effective gain drift compensation with simple circuitry. It was also shown that off-the-shelf temperature sensors can be used for the same purpose [173] (Fig. 18).

### Fast timing

The various signal multiplexing methods introduced in this review can significantly reduce the signal readout channels. However, the signal multiplexing accumulates the intrinsic capacitance and dark current of the SiPM. The undesirable parasitic detector capacitance of the SiPM not only degrades the SNR of detector signal output by providing a sinking path to the ground, but also retards the slope of the rising edge, which degrades the timing performance of SiPM-based detectors [40]. Baseline fluctuation due to the dark counts is another source of timing performance degradation.

#### Analog high pass filters

High-pass filtering of timing signals is a simple but effective method to improve the timing and count-rate performances of SiPM-based PET detectors. High-pass filters with pole-zero cancellation applied to the SiPM are useful for reducing the baseline fluctuation and pulse width of SiPM output signals; this led to improvements in the timing and count-rate performance [161, 174]. The usefulness of high-pass filtering for developing high-performance PET detectors while multiplexing SiPM signals was also demonstrated [94, 175].

#### Bootstrapping

**Fig. 19:**
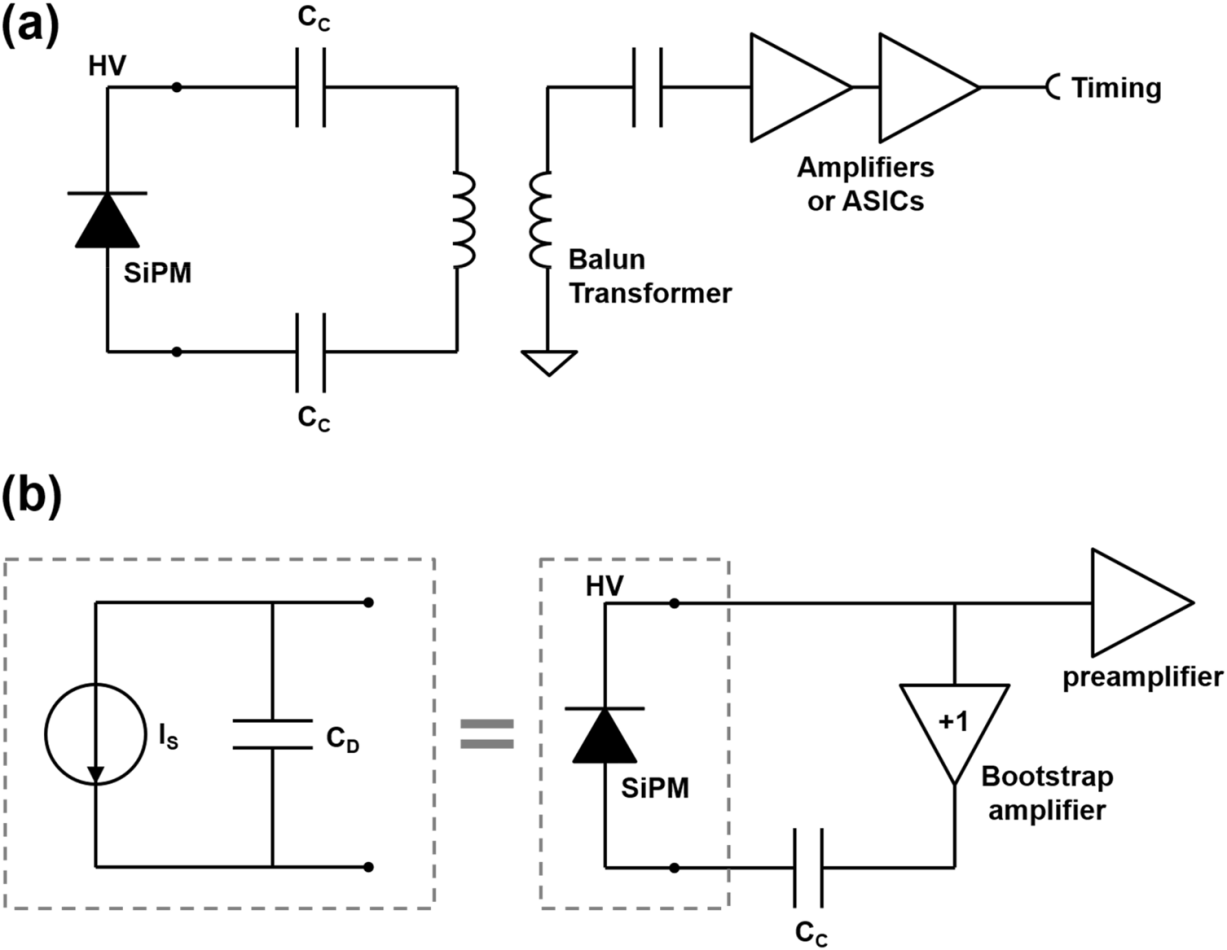
Bootstrapping methods for SiPM capacitance compensation: **a** passive method using a balun transformer and **b** active method using a bootstrapping amplifier

To address this timing performance degrading problem, capacitance compensation or “bootstrapping” techniques have been investigated. In passive bootstrapping compensation circuits [176–178] (Fig. 19a), the cathode and anode of the SiPM are connected to one end of a balun transformer, the other end of which is connected to a high-bandwidth amplifier. The balanced-to-unbalanced connection of the transformer with a 1:1 turn ratio should result in a twofold amplified signal output, yet the signal maximum amplitude was measured to be ~ 3.5, owing to a decreased effective terminal capacitance of the SiPM [176, 179]. However, the use of balun transformers and the high-power consumption of the high-speed amplifiers remain challenges in the implementation of this technique on an ASIC.

Miller effect was exploited to implement an active bootstrapping technique [180, 181]. A unity gain amplifier, or so called “bootstrapping” amplifier, was placed between the cathode and anode of the SiPM (Fig. 19b). With this “bootstrapping” amplifier, the feedback factor can be modeled as C_C_C_D_/(C_C_ + C_D_). If the DC blocking capacitor C_C_ is sufficiently larger than the detector capacitance C_D_, the feedback factor is approximately equal to C_D_. Then according to the Miller effect, the input capacitance C_in_ can be reformed as C_D_(1−A) which will be zero if a unity gain amplifier is used as the “bootstrapping” amplifier. So, the effective detector capacitance seen in the following front-end circuit was then reduced by the Miller effect while preventing any undesirable voltage signal or noise signal from entering the following circuits.

#### Cherenkov photon readout

Recent efforts to detect fast prompt photons, such as Cherenkov photons, have pushed the limits of timing performance in SiPM-based TOF PET detectors [178, 179, 182–186]. When estimating the interaction time of gamma rays based on the prompt photons, the following two points should be considered [184]: (1) SiPM and subsequent readout electronics should have a single-photon timing resolution that is as low as possible and a − 3 dB bandwidth with > 1 GHz sampling rate [178, 187], respectively; (2) Low noise level should be sufficiently low to avoid false triggers caused by the threshold equivalent to a few photon level [188]. Gundacker et al. [178] implemented a high-frequency readout electronics that utilize passive capacitance compensation and RF amplifiers to take full advantage of the SiPM with low single-photon timing resolution. The fast readout electronics with a − 3 dB bandwidth and ~ 1.5 GHz sampling rate yielded CTR values of 58 ± 2 ps and 98 ± 3 ps, respectively, by employing faint Cherenkov light from 2 × 2 × 3 mm to 2 × 2 × 20 mm LSO:Ce:Ca crystals coupled to FBK NUV-HD SiPMs. BGO crystals with dimensions of 2 × 2 × 3 mm also yielded a remarkable CTR value of 158 ± 3 ps. In other investigations with 3 × 3 × 3 mm BGO crystals [179, 183, 184], the capacitance compensated high-frequency SiPM readout electronics for measuring fast Cherenkov photons achieved timing resolution of 105–200 ps.

## Summary and conclusions

This paper provides a systematic review of the signal readout schemes for PET detectors based on SiPMs. Several different crystal-to-SiPM coupling and visible light photon readout schemes were introduced, and various signal multiplexing techniques that reduce the readout complexity of high-performance PET systems were reviewed. Technologies to improve the stability and timing performance of SiPM-based PET detectors were also discussed.

Most readout schemes for SiPM-based PET detectors have trade-offs between various factors (e.g. spatial, energy, and timing resolutions, signal crosstalk, manufacturing cost, size and complexity of the readout circuitry, etc.). As described throughout this review, recent research interest is not to focus on one factor, but rather to overcome the trade-offs and improve the imaging quality and overall performance of PET detectors.

## References

[1] Phelps ME (2000). Positron emission tomography provides molecular imaging of biological processes. P Natl Acad Sci.

[2] Pomper MG, Lee JS (2005). Small animal imaging in drug development. Curr Pharm Des.

[3] Margolis DJA (2007). Molecular imaging techniques in body imaging. Radiology.

[4] Anderson RC (2010). Management impact of Ga-68-DOTATATE PET/CT in neuroendocrine tumors. Nucl Med Mol Imaging.

[5] Anger HO, Gottschalk A (1963). Localization of brain tumors with the positron scintillation camera. J Nucl Med.

[6] Anger HO (1964). Scintillation camera with multichannel collimators. J Nucl Med.

[7] Phelps ME (1975). Application of annihilation coincidence detection to transaxial reconstruction tomography. J Nucl Med.

[8] Thompson CJ, Yamamoto Y, Meyer E (1976). A position imaging system for the measurement of regional cerebral blood flow. Appl Opt Instrum Med V.

[9] Williams CW, Crabtree MC, Burgiss SG (1979). Design and performance-characteristics of a positron emission computed axial tomograph: Ecat-Ii. IEEE Trans Nucl Sci.

[10] Karp JS (1997). Three-dimensional imaging characteristics of the HEAD PENN-PET scanner. J Nucl Med.

[11] Spinks TJ (2000). Physical characteristics of the ECAT EXACT3D positron tomograph. Phys Med Biol.

[12] Surti S (2007). Performance of philips gemini TF PET/CT scanner with special consideration for its time-of-flight imaging capabilities. J Nucl Med.

[13] Bettinardi V (2011). Physical performance of the new hybrid PET/CT discovery-690. Med Phys.

[14] Son JW (2017). Proof-of-concept prototype time-of-flight PET system based on high-quantum-efficiency multianode PMTs. Med Phys.

[15] Wagatsuma K (2017). Comparison between new-generation SiPM-based and conventional PMT-based TOF-PET/CT. Phys Med Eur J Med Phys.

[16] Gatti E, Svelto V (1966). Review of theories and experiments of resolving time with scintillation counters. Nucl Instrum Methods.

[17] Moorhead ME, Tanner NW (1996). Optical properties of an EMI K2CsSb bialkali photocathode. Nuclear Instrum Methods Phys Res Sect Accelerat Spectrom Detect Assoc Equip.

[18] Moszynski M, Bengtson B (1979). Status of timing with plastic scintillation detectors. Nucl Instrum Methods.

[19] Moszynski M (2004). New fast photomultipliers with a screening grid at the anode. IEEE Trans Nucl Sci.

[20] Schaart DR (2021). Physics and technology of time-of-flight PET detectors. Phys Med Biol.

[21] Britvitch I (2004). Avalanche photodiodes now and possible developments. Nuclear Instrum Methods Phys Res Sect Accelerat Spectrom Detect Assoc Equip.

[22] Budinger TF (1998). PET instrumentation: what are the limits?. Semin Nucl Med.

[23] Humm JL, Rosenfeld A, Del Guerra A (2003). From PET detectors to PET scanners. Eur J Nucl Med Mol Imaging.

[24] Wu Y (2009). A study of the timing properties of position-sensitive avalanche photodiodes. Phys Med Biol.

[25] Peng H, Levin CS (2010). Recent developments in PET instrumentation. Curr Pharm Biotechnol.

[26] Lee JS, Kim JH (2014). Recent advances in hybrid molecular imaging systems. Semin Musculoskelet Radiol.

[27] Auffray E (2006). First results with the clearpet small animal PET scanners. Radiat Detect Med Appl.

[28] Bergeron M (2009). Performance evaluation of the LabPET APD-based digital PET scanner. IEEE Trans Nucl Sci.

[29] Catana C (2008). Simultaneous in vivo positron emission tomography and magnetic resonance imaging. Proc Natl Acad Sci USA.

[30] Delso G (2011). Performance measurements of the siemens mMR integrated whole-body PET/MR scanner. J Nucl Med.

[31] Grazioso R (2006). APD-based PET detector for simultaneous PET/MR imaging. Nuclear Instrum Methods Phys Res Sect Accelerat Spectrom Detect Assoc Equip.

[32] Judenhofer MS (2008). Simultaneous PET-MRI: a new approach for functional and morphological imaging. Nat Med.

[33] Yoon HS (2012). Initial results of simultaneous PET/MRI experiments with an MRI-compatible silicon photomultiplier PET scanner. J Nucl Med.

[34] Yamamoto S (2012). Simultaneous imaging using Si-PM-based PET and MRI for development of an integrated PET/MRI system. Phys Med Biol.

[35] Ko GB (2016). Simultaneous multiparametric PET/MRI with silicon photomultiplier PET and ultra-high-field mri for small-animal imaging. J Nucl Med.

[36] Stillman G, Wolfe C (1977). Avalanche photodiodes. Semiconductors and semimetals.

[37] Renker D (2006). Geiger-mode avalanche photodiodes, history, properties and problems. Nuclear Instrum Methods Phys Res Sect Accelerat Spectrom Detect Assoc Equip.

[38] Renker D, Lorenz E (2009). Advances in solid state photon detectors. J Instrum.

[39] Acerbi F, Gundacker S (2019). Understanding and simulating SiPMs. Nuclear Instrum Methods Phys Res Sect Accelerat Spectrom Detect Assoc Equip.

[40] Gundacker S, Heering A (2020). The silicon photomultiplier: fundamentals and applications of a modern solid-state photon detector. Phys Med Biol.

[41] Lecoq P, Gundacker S (2021). SiPM applications in positron emission tomography: toward ultimate PET time-of-flight resolution. Eur Phys J Plus.

[42] Vinogradov S, Popova E (2020). Status and perspectives of solid state photon detectors. Nuclear Instrum Methods Phys Res Sect Accelerat Spectrom Detect Assoc Equip.

[43] Akram MSH (2017). MRI compatibility study of an integrated PET/RF-coil prototype system at 3T. J Magn Reson.

[44] Gonzalez AJ (2019). Initial Results of the MINDView PET Insert Inside the 3T mMR. IEEE Trans Radiat Plasma Med Sci.

[45] Hong SJ (2008). An investigation into the use of Geiger-mode solid-state photomultipliers for simultaneous PET and MRI acquisition. IEEE Trans Nucl Sci.

[46] Lee JS, Hong SJ (2010). Geiger-mode avalanche photodiodes for PET/MRI.

[47] Roncali E, Cherry SR (2011). Application of silicon photomultipliers to positron emission tomography. Ann Biomed Eng.

[48] Casey M, et al. A next generation SiPM based PET/CT system with improved time and spatial resolution. J Nucl Med. 2017; 58.

[49] Grant AM (2016). NEMA NU 2–2012 performance studies for the SiPM-based ToF-PET component of the GE SIGNA PET/MR system. Med Phys.

[50] Ko GB (2016). Evaluation of a silicon photomultiplier PET insert for simultaneous PET and MR imaging. Med Phys.

[51] Reddin JS, et al. Performance Evaluation of the SiPM-based siemens biograph vision PET/CT system. In 2018 Ieee nuclear science symposium and medical imaging conference proceedings (Nss/Mic), 2018.

[52] Son JW (2020). SimPET: a preclinical PET insert for simultaneous PET/MR imaging. Mol Imag Biol.

[53] Levin CS (2016). Design features and mutual compatibility studies of the time-of-flight PET capable GE SIGNA PET/MR system. IEEE Trans Med Imaging.

[54] van Sluis J (2019). Performance characteristics of the digital biograph vision PET/CT system. J Nucl Med.

[55] Seifert S (2009). Simulation of silicon photomultiplier signals. IEEE Trans Nucl Sci.

[56] Cherry SR, Dahlbom M, Phelps ME (2004). PET: physics, instrumentation, and scanners. PET.

[57] Kang HG, et al. Optimization of a light guide for high resolution PET detectors using GATE optical simulation. In 2018 Ieee Nuclear science symposium and medical imaging conference proceedings (Nss/Mic), 2018.

[58] Surti S (2000). Optimizing the performance of a PET detector using discrete GSO crystals on a continuous lightguide. IEEE Trans Nucl Sci.

[59] Yoshida E (2019). Four-layered DOI-PET detector with quadrisected top layer crystals. Nuclear Instrum Methods Phys Res Sect Accelerat Spectrom Detect Assoc Equip.

[60] Ko GB (2013). Development of a front-end analog circuit for multi-channel SiPM readout and performance verification for various PET detector designs. Nuclear Instrum Methods Phys Res Sect Accelerat Spectrom Detect Assoc Equip.

[61] Song TY (2010). A sub-millimeter resolution PET detector module using a multi-pixel photon counter array. Phys Med Biol.

[62] Du JW, Bai XW, Cherry SR (2019). Performance comparison of depth-encoding detectors based on dual-ended readout and different SiPMs for high-resolution PET applications. Phys Med Biol.

[63] Du JW (2021). Performance evaluation of dual-ended readout PET detectors based on BGO arrays with different reflector arrangements. Phys Med Biol.

[64] Kang HG (2015). A dual-ended readout detector using a meantime method for SiPM TOF-DOI PET. IEEE Trans Nuclear Sci.

[65] Kang HG (2020). Crystal surface and reflector optimization for the SiPM-based dual-ended readout TOF-DOI PET detector. Biomed Phys Eng Exp.

[66] Yang Q (2020). Performance of two depth-encoding dual-ended readout PET detectors based on SiPM arrays with the same pitch size and different active areas. J Instrum.

[67] Derenzo SE (1989). Initial characterization of a position-sensitive photodiode bgo detector for pet. IEEE Trans Nucl Sci.

[68] Ito M, Hong SJ, Lee JS (2011). Positron emission tomography (PET) detectors with depth-of-interaction (DOI) capability. Biomed Eng Lett.

[69] Mohammadi I (2019). Minimization of parallax error in positron emission tomography using depth of interaction capable detectors: methods and apparatus. Biomed Phys Eng Exp.

[70] Hong SJ (2008). Concept verification of three-layer DOI detectors for small animal PET. IEEE Trans Nuclear Sci.

[71] Lee MS, Lee JS (2015). Depth-of-interaction measurement in a single-layer crystal array with a single-ended readout using digital silicon photomultiplier. Phys Med Biol.

[72] Pizzichemi M (2019). On light sharing TOF-PET modules with depth of interaction and 157 ps FWHM coincidence time resolution. Phys Med Biol.

[73] Shibuya K, et al. Timing resolution improved by DOI information in an LYSO TOF-PET detector. In 2007 Ieee Nuclear science symposium conference record. 2007; 1–11: 3678–80.

[74] Shibuya K (2008). Timing resolution improvement using DOI information in a four-layer scintillation detector for TOF-PET. Nuclear Instrum Methods Phys Res Sect Accelerat Spectrom Detect Assoc Equip.

[75] Toussaint M (2019). Analytical model of DOI-induced time bias in ultra-fast scintillation detectors for TOF-PET. Phys Med Biol.

[76] Kwon SI (2019). Dual-ended readout of bismuth germanate to improve timing resolution in time-of-flight PET. Phys Med Biol.

[77] Cates JW, Levin CS (2018). Evaluation of a clinical TOF-PET detector design that achieves <= 100 ps coincidence time resolution. Phys Med Biol.

[78] Lee MS (2021). High-resolution time-of-flight PET detector with 100 ps coincidence time resolution using a side-coupled phoswich configuration. Phys Med Biol.

[79] Pourashraf S (2021). Scalable electronic readout design for a 100 ps coincidence time resolution TOF-PET system. Phys Med Biol.

[80] Yeom JY, Vinke R, Levin CS (2014). Side readout of long scintillation crystal elements with digital SiPM for TOF-DOI PET. Med Phys.

[81] Cates JW, Levin CS, Design concepts and characterization of a next generation clinical PET detector. In 2016 Ieee nuclear science symposium, medical imaging conference and room-temperature semiconductor detector workshop (Nss/Mic/Rtsd), 2016.

[82] Wu C, Lee MS, Levin CS, Neural network-based inter-crystal scatter event positioning in a PET system design based on 3D position sensitive detectors. In 2020 IEEE nuclear science symposium and medical imaging conference (NSS/MIC): p. 1–3.

[83] Lee S, Lee JS (2021). Inter-crystal scattering recovery of light-sharing PET detectors using convolutional neural networks. Phys Med Biol.

[84] Park H, Lee JS (2020). SiPM signal readout for inter-crystal scatter event identification in PET detectors. Phys Med Biol.

[85] Hunter WC, De Witt DQ, Miyaoka RS (2021). Performance characteristics of a dual-sided position-sensitive sparse-sensor detector for gamma-ray imaging. IEEE Trans Radiat Plasma Med Sci.

[86] Karakatsanis NA, Zein SA, Nehmeh SA, Positron emission tomography with sparse block rings and continuous bed motion. In 2019 Ieee nuclear science symposium and medical imaging conference (Nss/Mic), 2019.

[87] Salomon A, et al. Sparse crystal setting and large axial FOV for integrated whole-body PET/MR. In 2011 Ieee nuclear science symposium and medical imaging conference (Nss/Mic), 2011; 2521–3.

[88] Zein SA (2021). Monte carlo simulation of the siemens biograph vision PET with extended axial field of view using sparse detector module rings configuration. IEEE Trans Radiat Plasma Med Sci.

[89] Zhang J, Knopp MI, Knopp MV (2019). Sparse detector configuration in SiPM digital photon counting PET: a feasibility study. Mol Imag Biol.

[90] Goertzen AL (2016). First results from a high-resolution small animal SiPM PET insert for PET/MR imaging at 7T. IEEE Trans Nucl Sci.

[91] Kwon SI (2011). Development of small-animal PET prototype using silicon photomultiplier (SiPM): initial results of phantom and animal imaging studies. J Nucl Med.

[92] Lee MS (2017). Prototype pre-clinical PET scanner with depth-of-interaction measurements using single-layer crystal array and single-ended readout. Phys Med Biol.

[93] Mackewn JE (2015). PET performance evaluation of a pre-clinical SiPM-based MR-compatible PET scanner. IEEE Trans Nucl Sci.

[94] Kim KY (2021). Performance evaluation of SimPET-X, a PET insert for simultaneous mouse total-body PET/MR imaging. Mol Imag Biol.

[95] Basiladze S (2016). Application specific integrated circuits for ionizing-radiation detectors (review, part 1). Instrum Exp Tech.

[96] Anghinolfi F (2004). NINO: an ultra-fast and low-power front-end amplifier/discriminator ASIC designed for the multigap resistive plate chamber. Nuclear Instrum Methods Phys Res Sect Accelerat Spectrom Detect Assoc Equip.

[97] Corsi F (2009). ASIC development for SiPM readout. J Instrum.

[98] Fischer P (2009). Fast self triggered multi channel readout ASIC for time and energy measurement. IEEE Trans Nucl Sci.

[99] Ollivier-Henry N (2011). Design and characteristics of a multichannel front-End ASIC using current-mode CSA for small-animal PET imaging. IEEE Trans Biomed Circuits Syst.

[100] Rolo MD, et al. A 64-channel ASIC for TOFPET applications. In 2012 Ieee nuclear science symposium and medical imaging conference record (Nss/Mic), 2012; 1460–4.

[101] Shen W, et al. STiC: a mixed mode chip for SiPM ToF applications. In 2012 Ieee nuclear science symposium and medical imaging conference record (Nss/Mic), 2012; 877–81.

[102] Zhu X, et al. TIMPIC-II: the second version time-based-readout ASIC for SSPM based PET applications. In 2012 Ieee nuclear science symposium and medical imaging conference record (Nss/Mic), 2012; p. 1474–8.

[103] Comerma A, et al. FlexToT: current mode ASIC for readout of common cathode SiPM arrays. In 2013 Ieee nuclear science symposium and medical imaging conference (Nss/Mic), 2013.

[104] Piemonte C (2013). Performance of FBK SiPMs coupled to PETA3 read-out ASIC for PET application. Nuclear Instrum Methods Phys Res Sect Accelerat Spectrom Detect Assoc Equip.

[105] Sacco I, Fischer P, Ritzert M (2013). PETA4: a multi-channel TDC/ADC ASIC for SiPM readout. J Instrum.

[106] Chen HS, et al. A dedicated readout ASIC for time-of-flight positron emission tomography using silicon photomultiplier (SiPM). In 2014 Ieee nuclear science symposium and medical imaging conference (Nss/Mic), 2014.

[107] Ahmad S (2015). Triroc: a multi-channel SiPM read-out ASIC for PET/PET-ToF Application. IEEE Trans Nucl Sci.

[108] Dohle R, et al. Very compact, water-cooled SiPM module for PET/MRT Applications. In 2015 European microelectronics packaging conference (Empc), 2015.

[109] Di Francesco A (2016). TOFPET2: a high-performance ASIC for time and amplitude measurements of SiPM signals in time-of-flight applications. J Instrum.

[110] Sarasola I (2017). A comparative study of the time performance between NINO and FlexToT ASICs. J Instrum.

[111] Schug D, et al. Measurements with a PET coincidence setup based on the PETA5 ASIC and FBK RGB-HD SiPMs. In 2017 Ieee nuclear science symposium and medical imaging conference (Nss/Mic), 2017.

[112] Chen Y, Deng Z, Liu Y (2018). DIET: a multi-channel SiPM readout ASIC for TOF-PET with individual energy and timing digitizer. J Instrum.

[113] Orita T (2018). The current mode time-over-threshold ASIC for a MPPC module in a TOF-PET system. Nuclear Instrum Methods Phys Res Sect Accelerat Spectrom Detect Assoc Equip.

[114] Shen W (2018). A silicon photomultiplier readout ASIC for Time-of-flight applications using a new time-of-recovery method. IEEE Trans Nucl Sci.

[115] Gomez S, et al. A high dynamic range asic for time of flight PET with pixelated and monolithic crystals. In 2019 IEEE nuclear science symposium and medical imaging conference (Nss/Mic), 2019.

[116] Sanchez D (2022). HRFlexToT: a high dynamic range ASIC for time-of-flight positron emission tomography. IEEE Trans Radiat Plasma Med Sci.

[117] Schug D (2019). Initial measurements with the PETsys TOFPET2 ASIC evaluation kit and a characterization of the ASIC TDC. IEEE Trans Radiat Plasma Med Sci.

[118] Calo PP (2019). SiPM readout electronics. Nuclear Instrum Methods Phys Res Sect Accelerat Spectrom Detect Assoc Equip.

[119] Anger HO (1958). Scintillation camera. Rev Sci Instrum.

[120] Yamamoto S, Watabe H, Hatazawa J (2011). Performance comparison of Si-PM-based block detectors with different pixel sizes for an ultrahigh-resolution small-animal PET system. Phys Med Biol.

[121] Jeon SJ (2017). Position error correction using homography in discretized positioning circuit for gamma-ray imaging detection system. IEEE Trans Nucl Sci.

[122] Kim D, et al. An improved gamma interaction position estimation using deep neural networks for resistor based multiplexing circuit. In 2017 Ieee nuclear science symposium and medical imaging conference (Nss/Mic), 2017.

[123] Park H, Lee JS (2019). Highly multiplexed SiPM signal readout for brain-dedicated TOF-DOI PET detectors. Phys Med Eur J Med Phys.

[124] Seo M, Park H, Lee JS (2021). Evaluation of large-area silicon photomultiplier arrays for positron emission tomography systems. Electronics.

[125] Wang Q (2015). A compact high resolution flat panel PET detector based on the new 4-side buttable MPPC for biomedical applications. Nuclear Instrum Methods Phys Res Sect Accelerat Spectrom Detect Assoc Equip.

[126] Won JY (2021). Development and Initial results of a brain PET insert for simultaneous 7-Tesla PET/MRI using an FPGA-only signal digitization method. IEEE Trans Med Imaging.

[127] Xu TP, et al. Development of multi-channel fast sipm readout electronics for clinical TOF PET detector. In 2014 Ieee Nuclear Science Symposium and Medical Imaging Conference (Nss/Mic), 2014.

[128] Zhang XH, Qi YJ, Zhao CL (2012). Design and development of compact readout electronics with silicon photomultiplier array for a compact imaging detector. Chin Phys C.

[129] Olcott PD, et al. Charge multiplexing readout for position sensitive avalanche photodiodes. 2005 Ieee Nuclear Science Symposium Conference Record. 2005; 1–5: 2935–7.

[130] Popov V, Majewski S, Weisenberger A (2003). Readout electronics for multianode photomultiplier tubes with pad matrix anode layout. IEEE Nucl Sci Conf R.

[131] Siegel S (1996). Simple charge division readouts for imaging scintillator arrays using a multi-channel PMT. IEEE Trans Nucl Sci.

[132] Janecek M (2012). A high-speed multi-channel readout for SSPM arrays. IEEE Trans Nucl Sci.

[133] Ko GB, Lee JS (2015). Performance characterization of high quantum efficiency metal package photomultiplier tubes for time-of-flight and high-resolution PET applications. Med Phys.

[134] Park H, Ko GB, Lee JS, Hybrid charge division multiplexing method for SiPM-based high-resolution PET detectors. J Nucl Med. 2016; 57. 10.1088/1361-6560/aa6aea 28368851

[135] Stolin AV (2014). Evaluation of imaging modules based on sensl array SB-8 for nuclear medicine applications. IEEE Trans Nucl Sci.

[136] Raylman RR (2014). A large area, silicon photomultiplier-based PET detector module. Nuclear Instrum Methods Phys Res Sect Accelerat Spectrom Detect Assoc Equip.

[137] Kwon SI, Lee JS (2014). Signal encoding method for a time-of-flight PET detector using a silicon photomultiplier array. Nucl Instrum Meth A.

[138] Jung J (2021). A diode-based symmetric charge division circuit with grounding path to reduce signal crosstalk and improve detector performance. IEEE Trans Radiat Plasma Med Sci.

[139] Olcott PD (2005). Compact readout electronics for position sensitive photomultiplier tubes. IEEE Trans Nucl Sci.

[140] Wang YJ (2012). Design and performance evaluation of a compact, large-area PET detector module based on silicon photomultipliers. Nuclear Instrum Methods Phys Res Sect Accelerat Spectrom Detect Assoc Equip.

[141] Won JY (2020). Comparator-less PET data acquisition system using single-ended memory interface input receivers of FPGA. Phys Med Biol.

[142] Downie E, Yang X, Peng H (2013). Investigation of analog charge multiplexing schemes for SiPM based PET block detectors. Phys Med Biol.

[143] Sun X, Lou K, Shao Y, Capacitor based multiplexing circuit for silicon photomultiplier array readout. 2014 IEEE Nuclear Science Symposium and Medical Imaging Conference (NSS/MIC); 2014: p. 1–5.

[144] Olcott PD, Glover G, Levin CS (2013). Cross-strip multiplexed electro-optical coupled scintillation detector for integrated PET/MRI. IEEE Trans Nucl Sci.

[145] Du JW (2013). A simple capacitive charge-division readout for position-sensitive solid-state photomultiplier arrays. IEEE Trans Nucl Sci.

[146] Choe HJ (2017). Development of capacitive multiplexing circuit for SiPM-based time-of-flight (TOF) PET detector. Phys Med Biol.

[147] Kim H (2016). A silicon photo-multiplier signal readout using strip-line and waveform sampling for Positron Emission Tomography. Nuclear Instrum Methods Phys Res Sect Accelerat Spectrom Detect Assoc Equip.

[148] Won JY, Ko GB, Lee JS (2016). Delay grid multiplexing: simple time-based multiplexing and readout method for silicon photomultipliers. Phys Med Biol.

[149] Vinke R, Yeom JY, Levin CS (2015). Electrical delay line multiplexing for pulsed mode radiation detectors. Phys Med Biol.

[150] Kim KB (2020). Feasibility study of multiplexing method using digital signal encoding technique. Nucl Eng Technol.

[151] Mishra M (2018). Frequency domain multiplexing of pulse mode radiation detectors. Nuclear Instrum Methods Phys Res Sect Accelerat Spectrom Detect Assoc Equip.

[152] Wonders MA, Flaska M (2020). Characterization of a mixed-sinusoid multiplexing scheme with silicon photomultipliers and an inorganic scintillator. Nuclear Instrum Methods Phys Res Sect Accelerat Spectrom Detect Assoc Equip.

[153] Yoon HS, Lee JS (2014). Bipolar analog signal multiplexing for position-sensitive PET block detectors. Phys Med Biol.

[154] Jung J (2018). An improved time over threshold method using bipolar signals. Phys Med Biol.

[155] Degenhardt C, et al. The digital silicon photomultiplier: a novel sensor for the detection of scintillation light. In 2009 Ieee nuclear science symposium conference record; 2009 1–5: 2383–6.

[156] Gruber M, Investigations on applying the time-of-flight method using cost effective hybrid Cherenkov radiators/scintillators for positron emission tomography. 2018, Wien.

[157] Spanoudaki VC (2007). Use of single photon counting detector arrays in combined PET/MR: characterization of LYSO-SiPM detector modules and comparison with a LSO-APD detector. J Instrum.

[158] Zeng C (2021). Evaluation of a PET detector based on SiPMs and FPGA-only MVT digitizers. Nuclear Instrum Methods Phys Res Sect Accelerat Spectrom Detect Assoc Equip.

[159] Fujiwara T (2010). Multi-level time-over-threshold method for energy resolving multi-channel systems. IEEE Trans Nucl Sci.

[160] Cates JW, Bieniosek MF, Levin CS (2017). Highly multiplexed signal readout for a time-of-flight positron emission tomography detector based on silicon photomultipliers. J Med Imag.

[161] Ko GB, Lee JS (2017). Single transmission-line readout method for silicon photomultiplier based time-of-flight and depth-of-interaction PET. Phys Med Biol.

[162] Yamamoto S (2010). Development of a Si-PM-based high-resolution PET system for small animals. Phys Med Biol.

[163] Pepin CM (2004). Properties of LYSO and recent LSO scintillators for phoswich PET detectors. IEEE Trans Nucl Sci.

[164] Streun M (2003). Pulse shape discrimination of LSO and LuYAP scintillators for depth of interaction detection in PET. IEEE Trans Nucl Sci.

[165] Yi M, Lee JS (2022). A time-based single transmission-line readout with position multiplexing. Biomed Eng Lett.

[166] Kaplan A (2009). Correction of SiPM temperature dependencies. Nuclear Instrum Methods Phys Res Sect Accelerat Spectrom Detect Assoc Equip.

[167] Zhou A (2020). Study on the FPGA-based temperature compensation for the SiPM of CEPC ECAL prototype. J Instrum.

[168] Gil A, et al. Programmable power supply system for SiPM bias. 2011 Ieee nuclear science symposium and medical imaging conference (Nss/Mic), 2011: p. 787–90.

[169] Licciulli F, Indiveri I, Marzocca C (2013). A novel technique for the stabilization of SiPM gain against temperature variations. IEEE Trans Nucl Sci.

[170] Licciulli F, Marzocca C (2015). An active compensation system for the temperature dependence of SiPM gain. IEEE Trans Nucl Sci.

[171] Miyamoto H, et al. SiPM development and application for astroparticle physics experiments. In 31th International Cosmic Ray Conference, Łódz, Poland; 2009.

[172] Kuznetsov E (2018). Temperature-compensated silicon photomultiplier. Nuclear Instrum Methods Phys Res Sect Accelerat Spectrom Detect Assoc Equip.

[173] Shim HS, Park H, Lee JS (2021). A temperature-dependent gain compensation technique for positron emission tomography detectors based on a silicon photomultiplier. Phys Med Biol.

[174] Gola A, Piemonte C, Tarolli A (2013). Analog circuit for timing measurements with large area SiPMs coupled to LYSO crystals. IEEE Trans Nucl Sci.

[175] Bieniosek MF (2016). Analog filtering methods improve leading edge timing performance of multiplexed SiPMs. Phys Med Biol.

[176] Zhang N, Schmand, Bootstrapping readout for large terminal capacitance analog-SiPM based time-of-flight PET detector. 2018, Google Patents.

[177] Pourashraf S (2021). Investigation of electronic signal processing chains for a prototype TOF-PET system with 100 ps coincidence time resolution. IEEE Trans Radiat Plasma Med Sci.

[178] Gundacker S (2019). High-frequency SiPM readout advances measured coincidence time resolution limits in TOF-PET. Phys Med Biol.

[179] Cates JW, Levin CS (2019). Electronics method to advance the coincidence time resolution with bismuth germanate. Phys Med Biol.

[180] Kwon I (2015). Compensation of the detector capacitance presented to charge-sensitive preamplifiers using the Miller effect. Nuclear Instrum Methods Phys Res Sect Accelerat Spectrom Detect Assoc Equip.

[181] Kim D, Kim CH, Kwon I (2020). Experimental results on a detector capacitance compensation technique for multiplexing SiPM channels. Nuclear Instrum Methods Phys Res Sect Accelerat Spectrom Detect Assoc Equip.

[182] Kratochwil N, Gundacker S, Auffray E (2021). A roadmap for sole Cherenkov radiators with SiPMs in TOF-PET. Phys Med Biol.

[183] Gundacker S (2020). Experimental time resolution limits of modern SiPMs and TOF-PET detectors exploring different scintillators and Cherenkov emission. Phys Med Biol.

[184] Gonzalez-Montoro A (2022). Cherenkov radiation-based coincidence time resolution measurements in BGO scintillators. Front Phys.

[185] Dolenec R (2016). The performance of silicon photomultipliers in cherenkov TOF PET. IEEE Trans Nucl Sci.

[186] Consuegra D, et al, Improving the cherenkov based PET performance using multi-layer detectors*.* 2019 Ieee Nuclear Science Symposium and Medical Imaging Conference (Nss/Mic); 2019.

[187] Gundacker S (2016). Measurement of intrinsic rise times for various L(Y) SO and LuAG scintillators with a general study of prompt photons to achieve 10 ps in TOF-PET. Phys Med Biol.

[188] Gundacker S (2016). State of the art timing in TOF-PET detectors with LuAG, GAGG and L(Y)SO scintillators of various sizes coupled to FBK-SiPMs. J Instrum.

